# The kinetics of maternal and self-developed *Streptococcus suis*-specific antibodies

**DOI:** 10.1186/s40813-025-00422-z

**Published:** 2025-02-07

**Authors:** Sandra Vreman, Rutger Jansen, Mikael Bastian, Patricia Beckers, Miriam van Riet, Helmi Fijten, Jan Fledderus, Astrid de Greeff, Hélène Winkelman, Norbert Stockhofe-Zurwieden, Lluís Fabà, Henk J. Wisselink, Manouk Vrieling

**Affiliations:** 1 Wageningen Bioveterinary Research, Wageningen University & Research, P.O. Box 65, Lelystad, 8200 AB The Netherlands; 2 Boehringer Ingelheim Animal Health Netherlands B.V, Basisweg 10, Amsterdam, 1043 AP The Netherlands; 3 ForFarmers Nederland B.V, P.O. Box 91, Lochem, 7240 AB The Netherlands; 4 Trouw Nutrition, R&D, Swine Research Centre, 5831 JN, Veerstraat 38, Boxmeer, The Netherlands

**Keywords:** *Streptococcus suis*, Piglet, Sow, Antibodies, Colostrum, Immune gap, Maternal-derived antibodies (MDA), Field study

## Abstract

**Background:**

*Streptococcus suis (S. suis)* infections are responsible for a large disease burden in piglets during the nursery phase, compromising animal welfare and increasing antibiotic use. The immune gap caused by decreased maternal-derived antibodies (MDA) and insufficient levels or functionality of acquired antibodies in weaned pigs could play a role in the increased susceptibility to *S. suis* infections. To better understand this, two studies were performed. Study I evaluated the associations between sow antibodies in colostrum and serum, birth parameters (e.g., birth weight, colostrum intake and piglet growth) and the levels of *S. suis*-specific (serotypes 2 and 9) antibodies in one-day-old piglets from four farms. Subsequently, study II used one of these farms to evaluate *S. suis*-specific and total antibody kinetics in piglets (10 litters with 6 selected piglets per litter, total *n* = 60) from birth until 10 weeks of age. Additionally, tonsil swabs from sows and piglets were taken to evaluate the *S. suis* tonsillar carrier status (serotypes 2 and 9) before and after weaning.

**Results:**

High variability in serum and colostrum antibody levels was observed between and within the four farms (study I). In study II, there was a decrease in *S. suis-*specific MDA after 24 h of age, with the lowest level occurring at approximately 18/19 days of age. Afterwards, there was an increase in specific antibodies, most likely due to acquired immunity. Colostrum intake, birth weight and 24-h weight gain after birth were important parameters that were positively associated with *S. suis* antibody levels in piglets after birth but also affected these antibody levels at a later age. All the piglet tonsils were colonized with *S. suis* serotype 9 before weaning, while the prevalence of serotype 2 increased after weaning.

**Conclusions:**

Total Ig against *S. suis* in serum declined after birth and the lowest level was detected just before weaning. Farmers and veterinarians should focus on piglets with low birth weights and late-born piglets because these parameters reduce both the *S. suis*-specific MDA preweaning and the specific antibodies acquired postweaning. Colostrum intake and 24 h-weight gain also affect the level of *S. suis* specific antibodies on day 1.

**Supplementary Information:**

The online version contains supplementary material available at 10.1186/s40813-025-00422-z.

## Background

*Streptococcus suis (S. suis)* is an important pathogen in pigs, causing infections that lead to decreased performance and increased mortality [1, 2], with significant consequences for swine health, welfare, and porcine production worldwide. *S. suis* infections usually occur in piglets up to 10 weeks of age, especially around weaning, causing meningitis, arthritis, endocarditis, polyserositis with associated lameness, neurologic signs and/or sudden death [1, 3]. *S. suis* is a very diverse pathogen. Currently, 29 serotypes of *S. suis* are recognized based on their capsular polysaccharides (CPSs) surrounding the bacterium [4]. Although *S. suis* serotype 2 is most frequently isolated from clinical cases worldwide, the number of serotype 9 isolates from diseased pigs has increased substantially in Europe [5, 6]. Both adult and young pigs can carry *S. suis* in the nose, tonsils, and nasopharynx as well as in the genital and gastrointestinal tract [7, 8], and pigs are often colonized by more than one serotype [9, 10]. These carrier pigs are often the source of infection for young sensitive piglets [11], but sows may also infect their own litters with saliva [12], and transmission during birth or suckling has been reported [13]. Infections with *S. suis* are most prevalent in the nursery phase between 4 and 10 weeks of age [14], but the peak incidence may differ per country, management system, or farm.

The high incidence of *S. suis* infections in the nursery phase coincides with a gap in humoral immunity, when maternal-derived antibody (MDA) levels have decreased and the acquired antibody response is still in development [15–18]. Piglets are born without systemic circulating antibodies, and MDA are only transferred through the uptake of IgG via the intestine from colostrum within the first 24 h after birth [19]. These passively acquired antibodies enter the bloodstream of piglets and act as a protective shield throughout the body in the same way as actively produced antibodies [20]. However, *S. suis* specific MDA are only present for a limited time [21, 22]. Studies have shown that clinical signs of *S. suis* infection appear when the level of MDA is low [23, 24] and that the production of *S. suis* antibodies slowly increases at the end of the nursery phase, when opsonizing antibodies that play a role in protection against *S. suis* infection start appearing [21, 22, 25, 26]. More research on the role of *S. suis-*specific antibodies in sow serum and colostrum and piglet birth parameters, such as colostrum intake and *S. suis*-specific antibody levels, can contribute to a better understanding of the dynamics of MDA in piglets from birth toward the development of acquired antibodies against *S. suis*. In study I, we evaluated the associations of sow and piglet parameters around birth with total (IgA, IgM, and IgG) and *S. suis*-specific (serotypes 2 and 9) antibody levels (IgG + IgM) in one-day-old piglets on four farms in the Netherlands. Subsequently, in study II, we used one of these farms to evaluate the kinetics of *S. suis* serotypes 2 and 9 and total IgA, IgM, and IgG antibodies in piglets from birth to 10 weeks of age. Additionally, from sows and piglets before and after weaning, tonsil swabs were taken to evaluate the *S. suis* status (serotypes 2 and 9) and the development of tonsil colonization in piglets.

## Methods

### Experimental design

Study I was a cross-sectional study with samples for diagnostic purposes performed on four farrow-to-finish farms (A to D) in the Netherlands with a high incidence of *S. suis* in weaned piglets. The study was performed from 2018 to 2019. Within the two years preceding the sampling, the piglets on these farms suffered from clinical disease related to *S. suis* serotype 2 and 9 infections. Growth-promoting antibiotics were not used by any of the four farms. Metaphylaxis is not allowed under Dutch antibiotic treatment regulations, and therefore not used. On farm D, the sows were vaccinated with a farm specific vaccine which was produced by Ceva Biovac (Angers, France). The vaccine consisted of *S. suis* serotype 2 (2 × 2017 brain isolate), *S. suis* serotype 9 (2017 brain isolate), *Pasteurella multocida* (1 × 2015 lung isolate) and *Actinobacillus pleuropneumoniae* (2 × 2016 lung isolate) strains. All were isolated from diseased pigs from farm D. For use as a vaccine, whole cell cultures were prepared from these strains and subsequently killed using formalin and mixed with an oil-based adjuvant. The sows received a primary vaccination (2.0 mL IM) at 60 days of gestation, which was boosted (2.0 mL IM) at 90 days of gestation. Subsequent boostering was performed at 90 days of each following gestation. Sows on all four farms were fed a commercial pelleted diet and housed in individual farrowing crates compliant with national legislation for the housing of farrowing sows.

Farms were visited for *S. suis* problem-related consulting. From each farm, four to six sows that started their farrowing process during the routine consultancy were included in the study. On farms A, B and C, the selection of the desired number of farrowing sows was completed on one day; for farm D, this selection was completed in two days. The sows were observed during farrowing, and no additional help or care was given during birth of the piglets. Within 5 min after birth, the piglets were weighed. The time of birth, birth order and body weight at birth (BWb) were recorded. For identification of the piglets, numbered ear tags were used. For study I on farms A-D, a total of 20 sows and 23 piglets were included (Supplementary Table 1).

Immediately after the birth of the first piglet (< 10 min), a colostrum sample was collected from the first three cranial teats of the upper half of the udder of the sow. The sampled teats were cleaned with paper tissues to remove dust and debris. Teats were milked manually until a volume of 10 mL of colostrum was obtained. The colostrum was refrigerated immediately after collection, aliquoted and stored at -20 °C until analysis by ELISA. One day after birth, the piglets were weighed again to determine the 24-hour body weight gain after birth (average daily gain D1 (ADG D1)), and the time of weighing was recorded. The colostrum intake of the piglets was calculated based on the parameters BWb (kg), weight gain (WG, gram) and the duration of colostrum intake (D in minutes) using the Theil calculation with equation: -106 + 2.26 WG + 200 BWb + 0.111D-1414 WG/D + 0.0182 WG/BWb, as previously published [27]. Blood samples were taken from the selected sows and piglets, and the serum was stored at -20 °C until analysis by ELISA.

One year after the finalization of study I, study II started on Farm B. Among the four farms in study I, this farm was the best equipped to perform a field study with a longer duration and had enough sows for a high likelihood of 10 spontaneous farrows during one day (1165 head sow herds with 55 farrows per weekly batch). In this study, which lasted from March 2020 to May 2020, piglets were born by the natural onset of farrowing. The farm was visited during the day of most natural farrowing (day 115 of gestation, study day 0). Ten sows that were farrowing during that day were included in the study. A total of 149 piglets were delivered to these ten sows. As described previously, all the piglets were weighed directly after birth (BWb), birth order was documented, and a standard ear tag was used for identification. Directly after birth, the umbilical cord was squeezed to obtain a precolostral-fed blood sample of at least 0.5 mL which was successful for 104 out of the 149 piglets (69.7%). Colostrum samples were collected and processed as described earlier, and the colostrum intake of each pig was calculated using the Theil calculation [27].

To reduce the risk of preweaning mortality, piglets were included on day 1 in the study if their birth weights were above 1,100 g [28, 29] and if successful an umbilical cord blood sample (> 0.5 mL whole blood) could be obtained. From these piglets, six piglets per sow were selected for the study, which were equally divided among littermates from the first to the last born piglet. There was no selection for sex. During the suckling period, the piglets were weighed, and blood samples were taken at 1, 6, 13, 20, and 23 days of age and during the postweaning period at 27, 35, 42, 56, and 69 days of age. Pigs were weaned at the age of 23 days, and at weaning, the siblings were kept together in a pen. On day 1, blood samples were also taken from the ten selected sows. The serum of these sows and the serum of the piglets were stored at -20 °C until analysis by ELISA. During the study period (from birth until day 69), eight of the 60 piglets (13%) died between day 4 and day 50, which is in line with the average mortality rate (measured from birth until 69 days) on this farm (11.5%) and other farms [30–32]. During the study period, piglets in the trial were not treated with antibiotics.

Due to the fully closed nature of the pens, there was no direct contact between piglets from different litters before and after weaning, limiting the potential for *S. suis* to be carried over by direct pig-to-pig contact.

Tonsillar samples from the piglets were collected at 23, 34 and 69 days of age and from the sows at weaning. Surgical pliers were used to open the mouths of the pigs. The sows’ mouths were opened using a metal mouth gag. Tonsillar samples were obtained by rubbing an eSwab™ 480CE (Copan Diagnostics, Inc., Carlsbad, CA, USA) on the tonsillar surface for several seconds. The swabs were immediately placed in eSwab™ 480CE tubes containing liquid Amies transport medium and transported to the laboratory at ambient temperature, after which the DNA was isolated for qPCR as described below.

### Quantification of porcine IgA, IgM and IgG

Greiner MICROLON^®^600 high binding ELISA plates were coated overnight at RT with antibodies against porcine IgA, IgM and IgG diluted in carbonate-bicarbonate buffer (Sigma Aldrich C3041, Saint Louis, MO, USA); see Supplementary Table 2 for details about the antibodies and dilutions. Blocking was performed with PBS + 1% BSA at pH 7.2 for 1 h at RT. Dilutions of sow and piglet sera, colostrum and sera of umbilical cord blood samples were prepared in PBS and added to the wells. Bound antibodies were detected with isotype-specific conjugates (Supplementary Table 2), using tetramethylbenzidine (TMB) as substrate. Reactions were stopped after 10–15 min by the addition of 0.5 M H_2_SO_4,_ and extinctions (at 450 nm) were measured on a microplate reader. Primary and secondary antibody incubations were performed for 1 h at RT, and the wells were washed three times with PBS after both incubations. To quantify the amount of IgA, IgM and IgG, a reference pig serum (Bethyl RS10-107, Bethyl Laboratories Inc., Montgomery, Texas, USA) was diluted in PBS to stock solutions of 3.6 µg IgA/mL, 3.5 µg IgM/mL and 3.5 µg IgG/mL. Serial dilutions of the stock solution of the reference serum were measured in duplicate, and a standard curve was fitted using 4-parameter logistics with SoftMax Pro Software. The standard curve was used to interpolate the OD450 values of individual samples to concentrations relative to the standard curve (µg/mL). All sera and other matrices from Study I were tested in duplicate, and the mean was used in the calculations. For Study II, the sera and colostrum were analyzed as single samples. The optimal dilutions of the coating antibodies, the matrices, the conjugates and the positive internal control sera were determined during preliminary standardizations.

### Porcine antibodies against whole cells of *S. suis* serotypes 2 and 9

*S. suis* serotype 2 strain 10 [33] and serotype 9 strain 8067 [5] were grown overnight in Todd Hewitt broth (THB) at 37 °C without shaking. The next day, the bacterial cells were harvested, washed with PBS, and inactivated by treatment with 0.5% formaldehyde for 1 h at room temperature (RT) with intermittent gentle mixing. Inactivated cells were washed and resuspended in PBS. Greiner MICROLON^®^600 high binding ELISA plates were coated overnight at RT with approximately 1E6 CFU/well of inactivated *S. suis* serotype 2 or *S. suis* serotype 9 bacteria in PBS. Blocking was performed with PBS + 1% BSA at pH 7.2 for 1 h at RT. A series of dilutions of sow and piglet serum, colostrum and umbilical blood samples were prepared in PBS and added to the wells. Bound antibodies were detected with a 1:10,000 dilution of peroxidase (PO)-conjugated anti-porcine-IgL (WBVR, mouse antibody (MAb) clone 27.2.1; [34]) using tetramethylbenzidine (TMB) as a substrate. Reactions were stopped after 10–15 min by the addition of 0.5 M H_2_SO_4,_ and extinctions (at 450 nm) were measured on a microplate reader. Serum and secondary antibody incubations were performed for 1 h at RT, and the wells were washed three times with PBS after both incubations. Serum samples from pigs that survived an experimental infection with *S. suis* serotype 2 strain 10 or *S. suis* serotype 9 strain 8067 were used as positive controls for the serotype 2 and serotype 9 ELISAs, respectively. Serial dilutions of the positive control were measured in duplicate, and a standard curve was fitted using 4-parameter logistics with SoftMax Pro Software. The standard curve was used to interpolate the OD450 values of individual samples to concentrations relative to those of the positive control (% positivity). All sera from Study I and II were analyzed in duplicate, and the mean was used in the calculations. The optimal dilutions of the coating antibodies, the matrices, the conjugates and the positive internal control sera were determined during preliminary standardizations.

### Colonization of tonsils by *S. suis* serotypes 2 and 9

DNA was isolated from tonsil swabs. For this purpose, the eSwab™ 480CE, including swab and Amies medium, were thawed during 30 min. The swabs were individually vortexed for 20 s and the Amies medium was immediately used for DNA isolation. Prior to DNA isolation, the samples were treated with an enzyme mixture to lyse the bacterial cells. Then, 46 µL of lysing mixture containing 20 µL of lysozyme (100 mg/mL), 1 µL of mutanolysin (5000 U/mL) and 25 µL of protein kinase K (600 AU/mL, included in the Qiagen DNeasy Blood & Tissue Kit) was added to 154 µL of the swab sample. The samples were mixed by vortexing and incubated for 30 min at 37 °C. Then, 200 µL of AL buffer from the Qiagen Blood and Tissue Kit was added, and the samples were vortexed for 15 s and incubated for 30 min at 56 °C. Then, 200 µL of 100% ethanol was added, and the samples were vortexed for 15 s. Purification was continued from step 4 of the Qiagen DNeasy Blood & Tissue Kit manual. Purified DNA was eluted in 30 µL of SuperQ^®^ water.

The primers and probe sequences specific for the *cpsJ* locus of *S. suis* serotype 2 (*cps2J*) and the *cpsH* locus of *S. suis* serotype 9 (*cps9H)* have been previously described [35]. For each *cps2J* PCR or *csp9*H PCR a standard curve control was added, containing pUC57 plasmid with the specific fragment of the *cps2J* of *cps9H* gene. Standard curve controls of pUC57-*cps2J* and pUC57-*cps9H* consist of dilutions 1 × 10^− 3^ -1 × 10^− 9^ of a 10^0^ stock with a concentration of approximately 3.5 × 10^8^ copies/ µL. Standard curves of internal positive controls (IPC) of pUC57-*cps2J*-IPC and pUC57-*cps9H*-IPC consist of dilutions 1 × 10^− 4^ -1 × 10^− 8^ of a 10^0^ stock with concentration of approximately 3.5 × 10^8^ copies/ µL. The slope for the standard curves should lie between − 3.1 and − 3.5. Negative controls containing no DNA were also included. Each PCR sample had a final volume of 20 µL and contained: 10 µL of 2×Taqman Fast Universal PCR mix (Thermo Fisher Scientific), 1,8 µL of 10 pmol of forward primer (F-cps2J or F-cps9H), 1,8 µL of 10 pmol of reverse primer (R-cps2J or R-cps9H), 0,25 µL of test probe (FAM-cps 2 J or FAM-cps9H), and 0.25 µL of IPC probe (VIC-IPC_cps2J/cps9H). To the experimental samples 2,5 µL of DNA isolated from tonsil swabs was added or 2,5µL of the standard curve dilution or water for the standard and negative controls. One microliter of IPC DNA (1 × 10^− 7^ dilution of pUC57 -cps2J-IPC or pUC57- csp9H-IPC) was added to all samples except the IPC standard curve samples. The reactions were supplemented with SuperQ^®^ water (Merck Millipore) up to a volume of 20 µL.

PCR was performed on an ABI 7500 FAST real-time PCR system (Applied Biosystems). The PCR conditions were as follows: 5 min at 95 °C, 40×[15 s at 95 °C, 1 min at 60 °C], probe detection at FAM/VIC, and a QPCR cutoff of 0.1. The amplification curves were analyzed with the ABI 7500 2.3 software from Applied Biosystems. The uninhibited Ct for 1 × 10^− 7^ ng of pUC57- *cps2J* DNA and pUC57- *cps9H* DNA was between 30 and 31 in both PCRs. Ct values below 35 were considered positive.

### Statistical analysis

The ELISA data were log10 (×+1) transformed to obtain normally distributed data. Comparisons were performed between the four farms using ordinary one-way ANOVA with Tukey’s multiple comparisons test (GraphPad Prism 9, USA). Significance across farms was tested with a one-sample t test on the Pearson coefficients to determine whether these were significantly different from 0 (R) [36].

Linear-mixed model: (R, lme4 package). For each antibody (*S. suis* serotype 2 reacting antibody (Ss2Ab), *S. suis* serotype 9 reacting antibody (Ss9Ab) and IgG), a default “null hypothesis” model (referred to as *H0*) was used to assess the effect of age (see Supplemental file *H0* model):

## Results

### Study I (farms A-D): antibody levels in sows and their one-day-old piglets

**Fig. 1:**
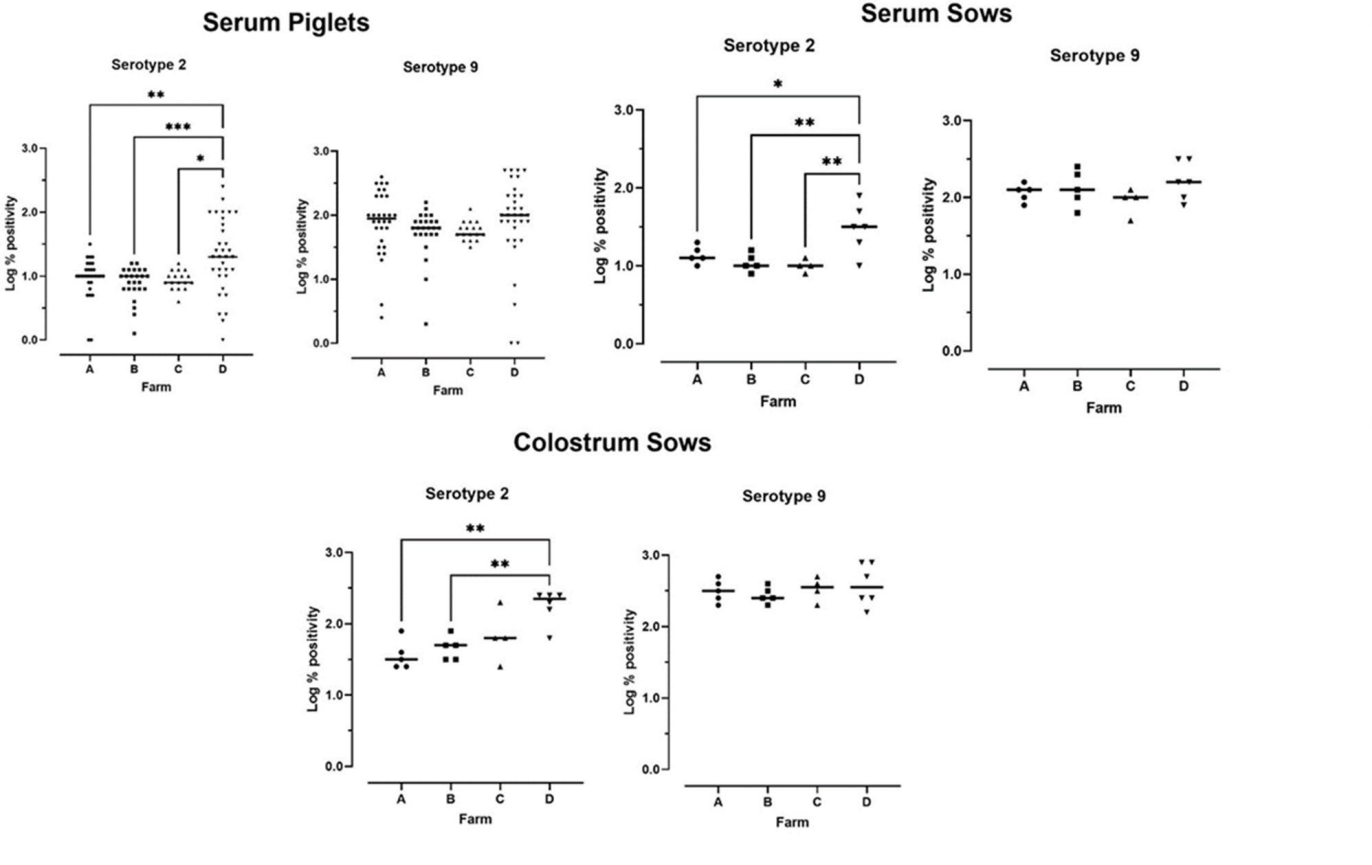
*S. suis*-specific antibodies in sows and piglets after birth. On four farms (**A** to **D**), *S. suis* serotype 2 and 9-reacting antibody levels (total Ig) were evaluated by ELISA in the colostrum and serum of sows at farrowing and in the serum samples of their offspring one day after birth. At each farm, four to six sows and five to six piglets per sow were selected. Antibody levels are expressed relative to a positive control serum (% positivity). Significant differences between farms are indicated by asterisks, with ns *P* ≥ 0.05, * *P* < 0.05, ** *P* < 0.01, *** *P* < 0.001, and **** *P* < 0.0001. Each dot shows the mean results of duplicate analyses of one animal. Joining lines showed significant differences between farms, for non-significant differences lines are not shown. The horizontal lines indicate the mean results per farm

First, we evaluated *S. suis*-specific and total antibody levels in the serum and colostrum of sows and in the serum of their one-day-old offspring on farms A to D. A high variability in serum and colostrum antibody levels was observed between and within the farms, but these differences were not significant (*P* > 0.05). On farm D, there was a significantly greater level of *S. suis-*serotype 2 reacting antibodies (Ss2Ab) in one-day-old piglets than in piglets from the other farms (Fig. 1). In addition, sows from this farm had a greater level of Ss2Ab in serum and colostrum than sows from the other three farms. There were no significant differences in *S. suis* serotype 9 reacting antibodies (Ss9Ab) between the different farms. Not all piglets on the same farm displayed uniform levels of *S. suis*-specific antibodies, as both piglets with high (% pos > 1) and low (% pos < 0.5) antibody levels were identified on the same farm. Total levels of IgG, IgA and IgM in colostrum and serum were also determined (Supplementary Fig. 1). Colostrum contained antibodies belonging to the three isotypes, with IgG displaying the highest absolute levels. The total IgA, IgG and IgM contents of the colostrum did not differ among the farms (*P* > 0.05). However, in the serum, higher total IgA levels were detected in sows and piglets from farm A than in those from the other farms (*P* < 0.05).

### Study I (farms A-D): correlation between birth parameters and *S. suis*-specific antibodies in one-day-old piglets

**Fig. 2:**
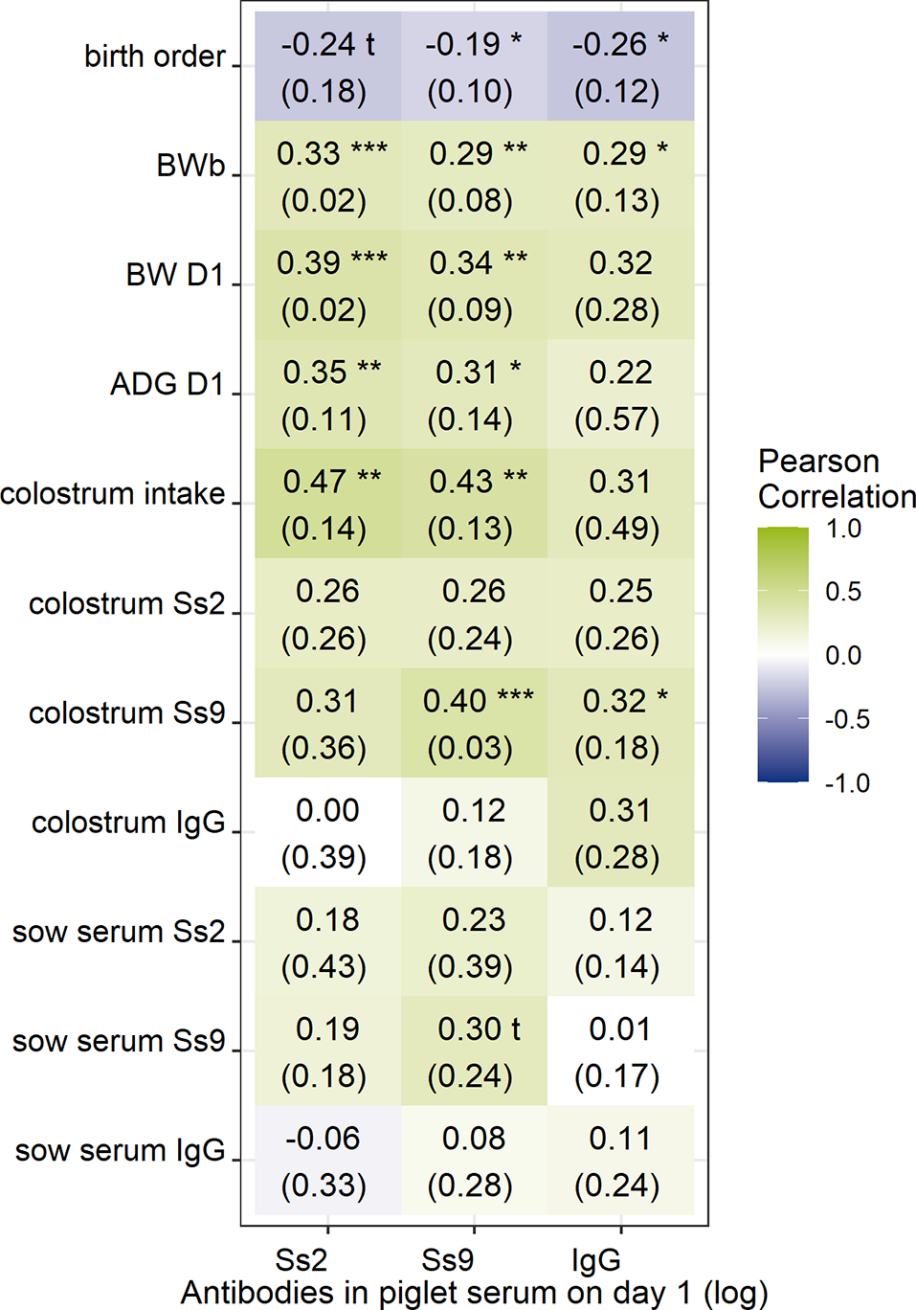
Correlation between sow and piglet parameters around birth for total *S. suis*-specific (serotypes 2 and 9) and total antibody levels in piglet serum one day after birth. The average Pearson correlation coefficient and STD (between brackets) of the coefficients across the four farms were calculated. A one-sample t test was performed to determine which correlation coefficients were significantly different from zero across the four farms. Significant differences are indicated by asterisks, with *P* < 0.1, * *P* < 0.05, ** *P* < 0.01, and *** *P* < 0.001. Blue indicates a negative correlation, and green indicates a positive correlation. The parameters used to determine the associations as described above were body weight at birth (BWb), body weight on day one (BW D1), average daily weight on day one (ADG D1), presence of *S. suis* serotype 2 (Ss2) and 9 (Ss9) reacting antibodies in colostrum, sow and pig serum, and estimated colostrum intake [27]

Piglet parameters around birth (birth order, colostrum intake, body weight at birth (BWb), weight on day 1 (BW D1), average daily weight gain (ADG) and total and specific antibody levels in sow colostrum and serum were analyzed for their associations with the level of *S. suis*-specific antibodies in one-day-old piglets on farms A to D. There was variability between the farms for the analyzed parameters (Supplementary Fig. 2). However, the average correlation across farms A-D (Fig. 2) showed that colostrum intake, birth weight, weight on day 1 and Ss9Ab level in colostrum had a significant positive association (*P* < 0.05) with the amount of *S. suis*-specific antibodies in one-day-old piglets. Birth order had a significant negative correlation, and later-born piglets had fewer *S. suis*-specific antibodies.

### Study II (farm B): kinetics of antibody levels in the serum of piglets

**Fig. 3:**
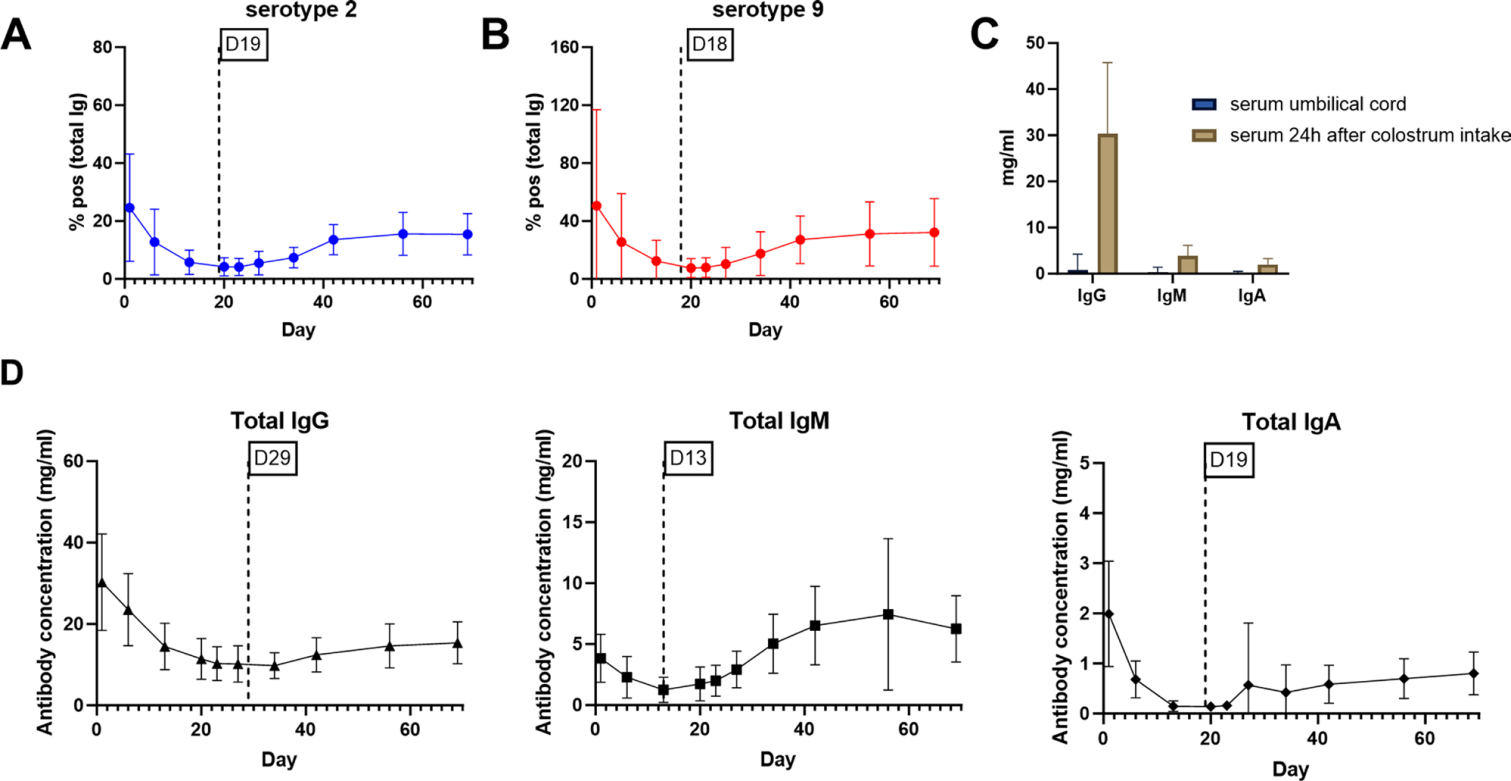
Kinetics of total antibodies and *S. suis*-specific antibodies in pigs from day 1 to day 69. Serum samples from six piglets per litter (ten litters in total) were analyzed by ELISA and mean results + SD are shown; (**A**) The level of *S. suis* serotype 2 reacting antibodies (total Ig) expressed relative to the positive control serum (% positivity); (**B**) The level of serotype 9 reacting antibodies (total Ig) expressed relative to the positive control serum (% positivity); (**C**) Total IgG, IgM and IgA antibody levels (mg/mL) in serum of umbilical cord blood (blue) (*n* = 39) in comparison to piglet serum 24 h after colostrum intake (brown) (*n* = 60); (**D**) Total IgG, IgM and IgA antibody levels (mg/mL) in piglet serum from day 1 to day 69

To understand how antibody levels in the serum of piglets change with age, *S. suis*-specific (IgG + IgM) and total IgA, IgG, and IgM antibody levels were evaluated in piglets from birth until 10 weeks of age. The levels of Ss2Ab and Ss9Ab after birth were greatest after colostrum intake, with high variability among the different litters. During the suckling period, the Ss2Ab and Ss9Ab levels rapidly decreased and reached their lowest points on days 19 and 18, respectively (Fig. 3A and B, Supplementary Fig. 3A and B). After weaning, the Ss2Ab and Ss9Ab antibody levels increased gradually. However, the overall level remained lower relative to the levels achieved immediately after colostrum intake. The umbilical cord blood samples, which represent the first blood sample of the piglet before colostrum intake, showed lower levels of IgG, IgM and IgA antibodies than did the serum samples collected directly after colostrum intake (Fig. 3C). Total antibodies, especially IgG, showed high levels directly after colostrum intake, followed by a decrease, with differences in the lowest calculated levels for IgG (29 days), IgM (13 days) and IgA (19 days) (Fig. 3D). In addition to the antibody kinetics, we evaluated the associations between birth parameters and *S. suis-*specific antibodies in one-day-old piglets as described in the previous paragraph. In studies I and II, this analysis showed a similar outcome for farm B; only in study II there was no significant positive correlation for the colostrum intake and a there was a negative correlation between sow serum IgG for the level of *S. suis*-specific antibodies in one-day-old piglets (Supplementary Fig. 4A).

### Study II: impact of birth parameters on *S. suis*-specific antibodies over time

**Fig. 4:**
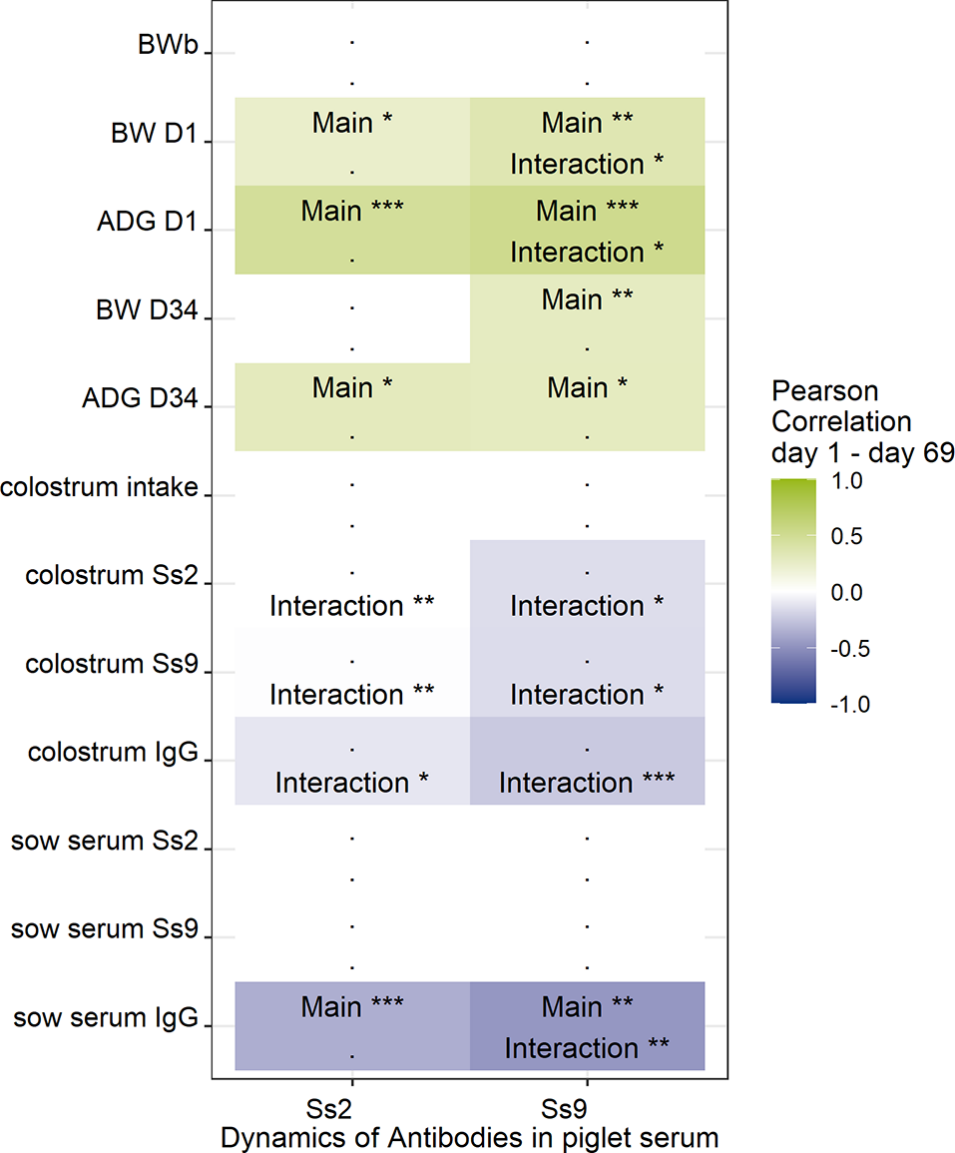
Influence of birth factors on *S. suis-*specific antibody dynamics in piglet serum. Linear mixed models were used to calculate potential carry-over effects from sow and birth factors until D69 (main effect) with or without a time interaction for Ss2Ab or Ss9Ab. When the average Pearson correlation coefficient at D1 and D69 is strong, the effect is green (positive) or blue (negative). When the correlation is significant but not strong, the effect is not colored; Inter (time interaction), Main (main effect)* *P* < 0.05, ** *P* < 0.01, *** *P* < 0.001; Body weight birth (BWb), body weight day one (BW D1); average daily day one (ADG D1); *S. suis* antibodies reacting with serotype 2 (Ss2) and serotype (Ss9); colostrum intake estimated on calculation Theil [27]

Linear mixed models were used to study potential carry-over effects from sow and birth factors until D69 (main effect) with or without a time interaction (variation in main effect over time) for Ss2Ab or Ss9Ab (Fig. 4 and Supplementary Fig. 5). Body weight gain-related parameters around birth (ADG D1, BW D1) and one week after weaning (ADG D34, BW D34) and the level of IgG in sow serum had a significant (*P* < 0.05) main effect on the kinetics of *S. suis*-specific antibodies. These findings indicate that the positive or negative effects of these parameters on the level of *S. suis* antibodies in the serum affect *S. suis* antibody levels throughout the entire 69-day study period. The ADG D1, BW D1, and sow serum IgG showed a main effect with a time interaction, indicating that the strength of the association of these factors with the level of *S. suis*-specific antibodies varied at different ages/study time points. For instance, ADG D1 was associated with increased Ss2Ab and Ss9Ab levels in piglet serum throughout the entire study period (Supplementary Fig. 5A). This suggests that piglets with a high increase in BW in the first 24 h after birth will have higher Ss2Ab and Ss9Ab levels in the first 69 days of life. For Ss2Ab, these effects were stable over time (no interaction); for Ss9Ab, there was a significant increase in the effect over time (interaction).

Most of the colostrum-related parameters showed clear changes over time (a time interaction). More precisely, the positive effect of colostrum antibodies and colostrum uptake seemed to decrease, and the effect at approximately day 69 was even negative (Supplementary Fig. 5D-F). In contrast, for the abovementioned effects of weight and colostrum composition on Ss2Ab and Ss9Ab serum levels in piglets, no parameters seemed to significantly affect IgG serum levels in piglets over time. In summary, weight and growth at D1 have a constant positive impact for the first 69 days of life, while colostrum composition mainly positively affects Ss2Ab/9 levels in the first weeks of life.

### Study II: *S. suis* serotypes 2 and 9 tonsil colonization

To evaluate tonsil *S. suis* colonization in sows and piglets over time, tonsil swabs were taken just before weaning (day 23) in piglets and sows and after weaning in piglets (days 34 and 69). Two out of nine sows (22%) tested positive for *S. suis* serotype 2, whereas six out of nine sows (67%) tested positive for *S. suis* serotype 9 (Table 1 and Supplementary Table 3). All serotype 2-positive sows were also positive for serotype 9. One sow was excluded from the analysis because we were unable to obtain a tonsil swab. Nearly all piglets (97%) and all litters tested positive for serotype 9 before weaning (day 23), while for serotype 2, only 26% of the piglets tested positive, and these animals were divided into 4/10 evaluated litters. After weaning, there was an increase in the number of serotype 2-positive piglets and litters, while there was no mixing of the piglets after weaning. On day 69, all the piglets were positive for serotype 9, and 81% of the piglets were positive for serotype 2. For serotype 9, there was no clear difference in the percentage of colonized bacteria before and after weaning.

**Table 1:** qPCR-positive tonsil swabs of *S. suis* serotypes 2 and 9 from pigs and sows

	Sow		Piglet					
Serotype	2	9	2			9		
Day (D)	D 23	D 23	D 23	D 34	D 69	D 23	D 34	D 69
p/total	2/9 **22%**	6/9 **67%**	15/58 **26%**	22/59 **37%**	43/54 **81%**	57/59 **97%**	56/59 **95%**	52/54 **96%**
Litters (p)	na	na	4	8	10	10	10	10

Ten sows were included in study II. From each sow, 6 pigs were selected and included in the study. Tonsillar swabs were taken from the sows and the piglets on the day of weaning (day 23) and from the pigs 34 and 69 days after birth. The swabs were tested for *S. suis* serotypes 2 and 9 by qPCR. Ct values less than 35 were considered positive (p)

## Discussion

Here, we investigated the transfer of *S. suis*-specific antibodies from sows to piglets 24 h after birth on four farms, and the MDA decrease and own acquired antibodies increase until 69 days of age, on one of these farms. Ss2Ab and Ss9Ab levels in piglets decreased just before weaning, which is most likely due to the decrease in serum MDA levels, as previously described [17, 18]. After this time point, there was a gradual increase in *S. suis*-specific antibodies until the end of the study, which likely represents the production of specific antibodies by the piglets following exposure to the bacterium. In the Netherlands, nearly all sows and piglets are colonized with *S. suis*, especially with serotype 9 [37, 38], as observed in this study. All the piglet tonsils were colonized with *S. suis* serotype 9 before weaning, while the prevalence of serotype 2 increased after weaning. The piglets most likely received the bacteria from their mother during birth or later via the saliva [12, 13]. This indicates that all sows, despite vaccination status, will transfer *S. suis*-specific antibodies to their offspring. In this study sows tested negative by PCR for the *S. suis* serotypes 2 and 9 (Table 1 and Supplemental Table 3). Piglets from PCR-negative sows tested finally PCR-positive without mixing the litter after weaning. This may indicate that besides transmission via the respiratory tract due to nose-nose contact between piglets [39], the environment, material and fomites possibly also played a role in transmission of the *S. suis* bacteria [35, 40]. This conclusion needs to be interpreted with care. Although the PCRs are thoroughly validated [10, 41] the PCR tests might be false negative due to limited test sensitivity or technical difficulties in taking proper tonsil swabs from adult sows. Furthermore, since vaginal and faecal swabs of sows were not tested, it cannot be excluded that piglets were colonized during birth or via faeces [6, 42].

The kinetics of the *S. suis*-specific antibodies in the piglets that we observed in this study were in line with the findings of a recent study on maternal immunity in piglets after sow vaccination with *S. suis* autogenous bacterins [21]. In that study, *S.suis*-specific antibody levels (IgG + IgM) were greater in seven-day-old piglets from vaccinated sows than in those from nonvaccinated sows. However, the levels rapidly decreased to their lowest point at 18 days of age. This dip was the same as that in the piglets of vaccinated and nonvaccinated sows, indicating that vaccination did not result in sustained MDA levels in post-weaned piglets. Interestingly, *S. suis* (auto) vaccination of sows can result in a lower disease incidence after weaning in nonvaccinated piglets with or without a clear presence of MDA [22, 25]. This suggests that in addition to MDA, other parts of the immune system are activated for protection at a later age, e.g., the transfer of pathogen-specific T cells by colostrum [43, 44]. In two other studies with nonvaccinated control piglets from nonvaccinated sows, the lowest IgG + IgM antibody levels were measured at approximately 5–7 weeks of age [22, 24, 45], indicating that the onset of antibody production by weaner piglets can occur later than we observed in our study. We were not able to differentiate between *S. suis* directed IgG and IgM. Others have shown that anti-capsule antibodies present before 4 weeks of age are mostly of the IgG isotype, and that piglets start to produce IgM antibodies around 6–10 weeks of age [16, 26]. Only after 7.5 weeks of age were antibodies present in blood of a sufficient level and functionality to aid in the killing of *S. suis* [16]. Altogether, these data indicate that there may be several weeks in the nursery phase when humoral immunity against *S. suis* is not optimal [3].

Several studies with a focus on the transfer of different pathogen-specific MDA from sows to offspring have shown that differences in the duration of persistence of MDA in offspring are mostly associated with the immunization or vaccination status of the sow. For example, Lauritsen et al. [46] showed that piglets that suckled infected sows were partially protected against infection with *Mycoplasma hyosynoviae* when challenged at 4.5 weeks of age, with indications that this was related to the MDA in colostrum. MDA persisted in the offspring of sows vaccinated with an inactivated Seneca virus vaccine A (SVA) until 42 days after a single vaccination and 90 days after a booster vaccination [47]. Another study highlighted the importance of MDA for early-life hepatitis E infections and showed that from 5 weeks of age, there is a rapid decline [48]. Overall, MDA play an important role in the prevention of early-life infections, considering that there are differences between pathogens and farm conditions. Importantly, MDA can also interfere with the development of acquired antibodies [18], e.g. presence of MDA was shown to impair the active antibody response to primary infection with Influenza [49]. How MDA affect antibody development after natural exposure to *S. suis* has not been studied, but there is a possibility that the quantity and quality of the response is influenced by the presence of MDA early in life.

We determined the total and *S. suis*-specific antibody levels in one-day-old piglets on four farms and investigated which parameters around birth influenced these levels. One of the farms (Farm D) had more *S. suis* serotype 2-reacting antibodies in both sows and piglets than the other farms. This farm applied autovaccination against serotypes 2 and 9 in sows, while the other farms did not. The immunization of sows or the greater disease pressure on farm D could explain these higher specific antibody levels [21, 50, 51]. We showed that a high level of *S. suis*-specific antibodies in colostrum results in a greater level of *S. suis*-specific antibodies in the serum of one-day-old piglets. Additionally, colostrum intake, birth weight and 24-h weight gain after birth and birth order are important parameters related to increased levels of *S. suis*-specific antibodies in the serum of one-day-old piglets, while total IgG in colostrum seems less important. For the colostrum intake the four different farms (A-D) in study I showed a significant correlation or at least a trend for the correlation between colostrum intake and *S. suis* specific antibodies in the serum of one-day-old piglets. In study II, we were unable to show this correlation for colostrum intake for farm B. However, the 24-h weight gain, an important parameter in the calculation of colostrum intake [27], was positively correlated with *S. suis* specific antibodies on farm B in both studies.

Several researchers have confirmed that sow and piglet factors at birth have an impact on the vitality of the piglets at birth and on survival and growth until the end of the nursery phase. Low birth weight piglets grow more slowly, are more likely to die before weaning [52, 53] and have lower colostrum intake and serum IgG levels at 24 h after birth [54]. Furthermore, birth order significantly affects the vitality of the piglets at birth; the later the piglets are born in a litter, the greater the risk of being stillborn or being disadvantaged with regard to colostrum intake, growth and survival [55]. Therefore, parameters influencing neonatal vitality and related to colostrum intake like birth order, body weight and body weight gain in the first 24 h after birth are important for total antibody development in piglets and are therefore important for the immune status and development of newborn piglets [56, 57]. However, the specific role of colostrum intake, birth parameters and body weight on the risk of development of *S. suis* infections later in life is unclear, as disease is also seen in well-developed piglets (Gottschalk personal communication).

There was a high variability among the four farms, and the correlations between birth parameters and piglet *S. suis*-specific antibody levels were not always consistent. Apparently, farm differences, such as genetic background, parity of sows used, and ambient temperature, may be as relevant as the sow-to-piglet relationship in affecting colostrum quality and immunoglobulin transfer [58–60]. Differences in porcine breeds, management and vaccination status on farms A-D could have contributed to this difference.

In this study, we evaluated the quantity of total (IgG, IgM and IgA) and *S. suis*-specific antibodies (IgG/IgM) in colostrum and serum. In both the serum and colostrum, IgG was the most abundant antibody and constituted approximately 90% of the total antibodies in the serum and 80% of the total antibodies in the colostrum. The four farms showed a variation in the levels of total antibodies, and farm A showed consistently higher IgA levels in the serum of piglets and sows, which can be important for mucosal immune responses in neonates [29]. We speculate that on this farm, the higher IgA levels could be related to prior respiratory or intestinal disease, resulting in a more activated mucosal immune system in the sows. It is known that colostrum contains mainly IgG. Within the transition toward milk, the IgG proportion changes dramatically in colostrum: 12 h after birth, there is a 50% reduction in IgG and an almost null amount of IgG within colostrum/milk by 24 h [58]. Moreover, the 24-hour weight gain, which is primarily due to colostrum intake, occurs for approximately 93% of the gain within the first 12 h after the first piglet is born [61], which enables the piglet to ingest colostrum with a high IgG concentration and start off with high IgG levels in the serum. When piglets grow rapidly, there is a dilution of total serum IgG in the whole body, resulting in a decrease in antibodies, as observed in our study and other studies [54, 62, 63]. The breakdown of MDA over time also contributes to the decline in IgG [64]. Studies have shown that high levels of IgG within the first week of life are positively related to the plasma concentration at 28 days of age [63, 65].

Finally, it is important to mention that the amount of *S. suis*-specific antibodies was only a minimal proportion of the total antibodies. To measure total IgG antibodies, we diluted the serum 20,000 times, and for the *S. suis*-specific antibody ELISA, we diluted the serum only 160 times. In addition to the amount of antibodies, their functionality is an important parameter. The capacity of *S. suis* antibodies to opsonize bacteria in vitro for phagocytes is an important measure of the protective immunity induced by a vaccine [21]. Furthermore, colostrum contains many other components, e.g., lipids, proteins, carbohydrates and leucocytes [60], which are essential for the growth and survival of piglets [66]. These components can differ between sows, which could lead to differences in the physical condition of the piglets and their growth [58]. Therefore, future studies will benefit from tests assessing the opsonizing capacity of *S. suis*-specific antibodies and from a more in-depth analysis of other functional components of colostrum, e.g., T cells and cytokines.

## Conclusions

There was a gap in the level of total Ig antibodies against *S. suis* between 3 and 6 weeks of age. Total Ig against *S. suis* in serum declined after birth and reached their lowest point before weaning (18–19 days after birth), after which self-developed antibodies gradually appeared and reached their highest level at 50–60 days of age. The most relevant and predictive factors for *S. suis*-specific antibody levels in one-day-old piglets were birth weight, birth order, colostrum intake and *S. suis* antibody level in colostrum, while total sow antibody levels in serum and colostrum were less predictive. In practice, low-birth-weight piglets, late-born piglets and piglets with insufficient 24 h-weight gain should receive extra attention because these piglets have reduced MDA and self-developed antibodies over time.

## Electronic supplementary material

Below is the link to the electronic supplementary material.

Supplementary Material 1

Supplementary Material 2

Supplementary Material 3

## Data Availability

Data is provided within the manuscript or supplementary information files.
