# Supplementary material for: The kinetics of maternal and self-developed Streptococcus suis-specific antibodies
Source: Porcine Health Manag. 2025 Feb 7;11:7. doi: 10.1186/s40813-025-00422-z (PMC11806565; doi:10.1186/s40813-025-00422-z)
Supplement: Supplementary file 1 — Supplementary Material 1 [file 40813_2025_422_MOESM1_ESM.pdf]

Supplemental Table 1 Description of the four pig farms (A to D) and number of animals in study comparing four farms.

Farm	Description of farms		Number of animals in study	
	No. of sows	Breeding Column	Sows	Pigs per litter
A	700	Rotation x Pietrain	5	6
B	1150	Topigs Norsvin 70 x Pietrain	5	5
C	650	Rotation x Pietrain	4	6
D	725	Topigs Norsvin 70 or 20 x Tempo	6	6

Supplemental Table 2: Coating antibodies, matrices and conjugates used for ELISAs to quantify porcine IgA, IgM and IgG

Detection of	Coating antibody	Matrix	Matrix dilution	Conjugate	Conjugate dilution
Pig IgA	anti-porcine-IgA (WBVR, MAb clone 27.9.1)	Serum sows	20.000	goat anti-porcine-IgA HRP conjugate A100-102P (Bethyl Laboratories Inc., Montgomory, TX, USA)	50.000
		Serum piglets	20.000		
		Colostrum	100.000		
		Umbilical cord blood	5000x		
Pig IgM	anti-porcine-IgM (WBVR, MAb clone 28.4.1)	Serum sows	20.000	goat anti-porcine IgM HRP conjugate A100-117P (Bethyl Laboratories Inc., Montgomory, TX, USA)	30.000
		Serum piglets	20.000		
		Colostrum	100.000		
		Umbilical cord blood	5000x		
Pig IgG	anti-porcine-IgG (WBVR, MAb clone 23.3.1a)	Serum sows	40.000	anti-porcine-IgL (WBVR, MAb 27.2.1-PO)	10:000
		Serum piglets	40.000		
		Colostrum	200.000		
		Umbilical cord blood	5000x		

Supplemental Table 3 *S. suis* serotype 2 and 9 qPCR on tonsil swabs of pigs and sows.

Number of animals tested positive in *S. suis* qPCR								
Litter	Sow		Piglets					
	Serotype 2	Serotype 9	Serotype 2			Serotype 9		
	Day 23	Day 23	Day 23	Day 34	Day 69	Day 23	Day 34	Day 69
1	1/1	1/1	4/6	6/6	6/6	6/6	6/6	6/6
2	ND	ND	0/5	1/5	4/5	5/5	5/5	5/5
3	0/1	0/1	1/6	1/6	4/4	6/6	6/6	4/4
4	0/1	1/1	0/6	2/6	5/6	6/6	6/6	6/6
5	0/1	1/1	0/6	0/6	2/5	4/6	4/6	3/5
6	0/1	0/1	0/6	0/6	4/6	6/6	6/6	6/6
7	0/1	1/1	0/6	2/6	5/6	5/6	5/6	5/6
8	0/1	0/1	6/6	6/6	4/4	6/6	6/6	4/4
9	0/1	1/1	4/6	3/6	6/6	6/6	5/6	6/6
10	1/1	1/1	0/5	1/5	4/5	5/5	5/5	5/5

Total pigs	2/9	6/9	15/58	22/59	43/54	57/59	56/59	52/54
	22%	67%	26 %	37%	81%	97%	95%	96%
Total litters positive	NA	NA	4	8	10	10	10	10

ND: Not determined; NA: Not applicable

Ten sows were included in study II. From each sow, 6 pigs were selected and included in the study. Tonsillar swabs were taken from the sows and the piglets on the day of weaning (day 23) and from the pigs 34 and 69 days after birth. The swabs were tested for *S. suis* serotypes 2 and 9 by qPCR. Ct values less than 35 were considered positive (p).
