# Supplementary material for: The kinetics of maternal and self-developed Streptococcus suis-specific antibodies
Source: Porcine Health Manag. 2025 Feb 7;11:7. doi: 10.1186/s40813-025-00422-z (PMC11806565; doi:10.1186/s40813-025-00422-z)
Supplement: Supplementary file 2 — Supplementary Material 2 [file 40813_2025_422_MOESM2_ESM.pdf]

H0 Model:

$$\log(\text{Ig} + 1) = \beta_0 + \beta_1 \text{poly}(\text{age}, 4) + \gamma_{0i} + \gamma_{1i} \text{poly}(\text{age}, 4) + \epsilon$$

Where

- β are fixed effect coefficients,
- γ are random effects coefficients for each i piglet,
- poly stands a polynomial of degree 4,
- ϵ is the residual error.

This H0 model fit the effect of age within each piglet (random effect), allowing us to extract the main effect of age regardless of the piglet, akin to the standard dynamics curve of the antibodies.

To test for the effect of each parameter (e.g. litter order, colostrum Ig, etc.), two other linear mixed models - a "Main effect" model and an "Interaction" model – were fitted incrementally, each building on top of the default H0 model and compared to it.

"Main effect" Model:

$$\log(\text{Ig} + 1) = \beta_0 + \beta_1 \text{poly}(\text{age}, 4) + \beta_2 \text{param} + \gamma_{0i} + \gamma_{1i} \text{poly}(\text{age}, 4) + \epsilon$$

Where

- β are fixed effect coefficients,
- γ are random effects coefficients for each i piglet,
- poly stands a polynomial of degree 4,
- param stands for parameter,
- ϵ is the residual error.

Meaning that on top of the main effect of age, a possible main effect of each parameter was also fitted, in order to assess an effect of this parameter on the antibody of interest. This "Main effect" model had no [parameter × age] interaction component, meaning that if there was a main effect, it was modeled to be the same at all ages across the 69-day periods.

In contrast, the "Interaction" model assessed the significance of an interaction between the effect of age and the effect of the parameter, in addition to the main effect of the parameter and the effect of age.

"Interaction" Model:

$$\log(\text{Ig} + 1) = \beta_0 + \beta_1\text{poly}(\text{age}, 4) + \beta_2\text{param} + \beta_3[\text{poly}(\text{age}, 4) \times \text{param}] + \gamma_{0i} + \gamma_{1i}\text{poly}(\text{age}, 4) + \epsilon$$

Where

- β are fixed effect coefficients,
- γ are random effects coefficients for each i piglet,
- poly stands a polynomial of degree 4,
- param stands for parameter,
- poly() x param stands for the interaction between the polynomial and the parameter,
- ϵ is the residual error.

The three models were compared to each other with Likelihood ratio test: a significant difference between the "main effect" model and the H0 model indicated that the parameter had an effect across the 69 days periods on the antibody variable of interest (Table 1). Similarly, a significant difference between the "interaction" model, and either the "main effect" or the H0 models indicated that an interaction between the parameter and the effect of age was supported by the data. Conversely, an absence of difference between the "interaction" model and the "main effect" model indicated that the complexity of an interaction was not supported by the data, hence confirming the conclusion that the main effect did not vary as a function of age.

Table 1 Interpretation table of the model comparisons

**Main > H0**	**Interaction > H0**	**Interaction > main**	**Interpretation**
no	no	no	Parameter had no significant impact on the Ig
yes	no	no	Parameter had an effect on the Ig that was independent of age.

no	yes	no	Parameter had an effect on the Ig that depended of age, but no “consistent” effect in the whole period.
no	no	yes	Unlikely to happen, since one would also expect a significant Interaction > H0 if the Interaction > Main is already significant.
yes	no	yes	
yes	yes	no	Parameter had an effect on the Ig that was independent of age.
no	yes	yes	Parameter had an effect on the Ig that depended of age, but no “consistent” effect in the whole period.
yes	yes	yes	Parameter had a main effect in the whole period that did depend on age.

Three models were built for each analysis and compared to one another. “H0” refers to the model with the time polynomials, “Main” to the model were the Parameter was added to the “H0” model, and “Interaction” to the model were the interaction between Parameter and the time polynomials was added to “Main” model. The first three columns compare whether more complex models improve on simple models: “yes” means the complex model is significantly better at alpha .05, no means the model is not significantly better. The interpretation is given for each combinations of model comparison.
