# Supplementary material for: The kinetics of maternal and self-developed Streptococcus suis-specific antibodies
Source: Porcine Health Manag. 2025 Feb 7;11:7. doi: 10.1186/s40813-025-00422-z (PMC11806565; doi:10.1186/s40813-025-00422-z)
Supplement: Supplementary file 3 — Supplementary Material 3 [file 40813_2025_422_MOESM3_ESM.docx]

**
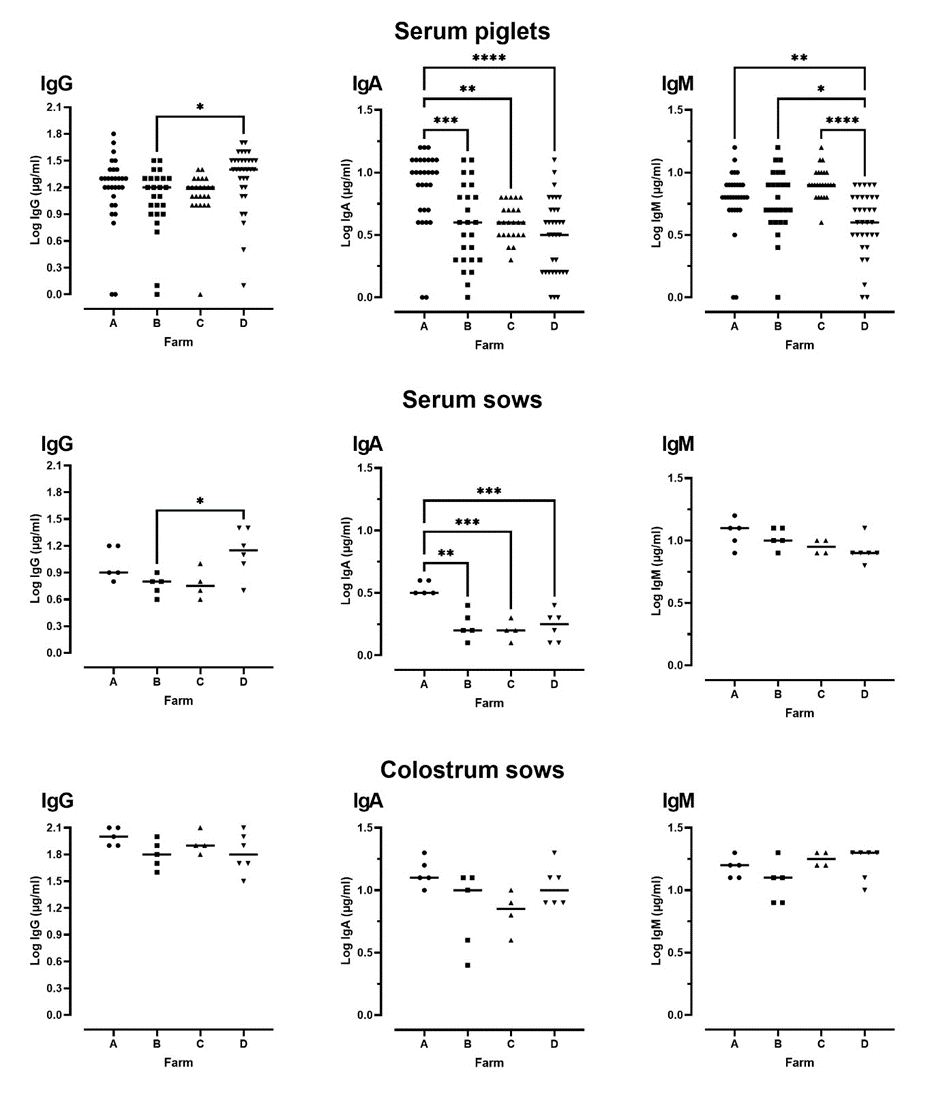
Supplemental Figures**

**Figure 1 Total antibody levels in sow and piglets around birth.** On four farms (A to D) total IgA, IgM and IgG levels were evaluated (ELISA) in colostrum and serum of sows at farrowing and in serum samples of their offspring, one day after birth. Per farm, four to six sows and five to six piglets per sow were selected. Significant differences are indicated by asterisks, with ns P ≥ 0.05, * P < 0.05, ** P < 0.01, *** P < 0.001, **** P < 0.0001. Each dot shows the mean result of duplicate analysis of one animal. Joining lines showed significant differences between farms, for non-significant differences lines are not shown. Horizontal lines indicate the mean results per farm.


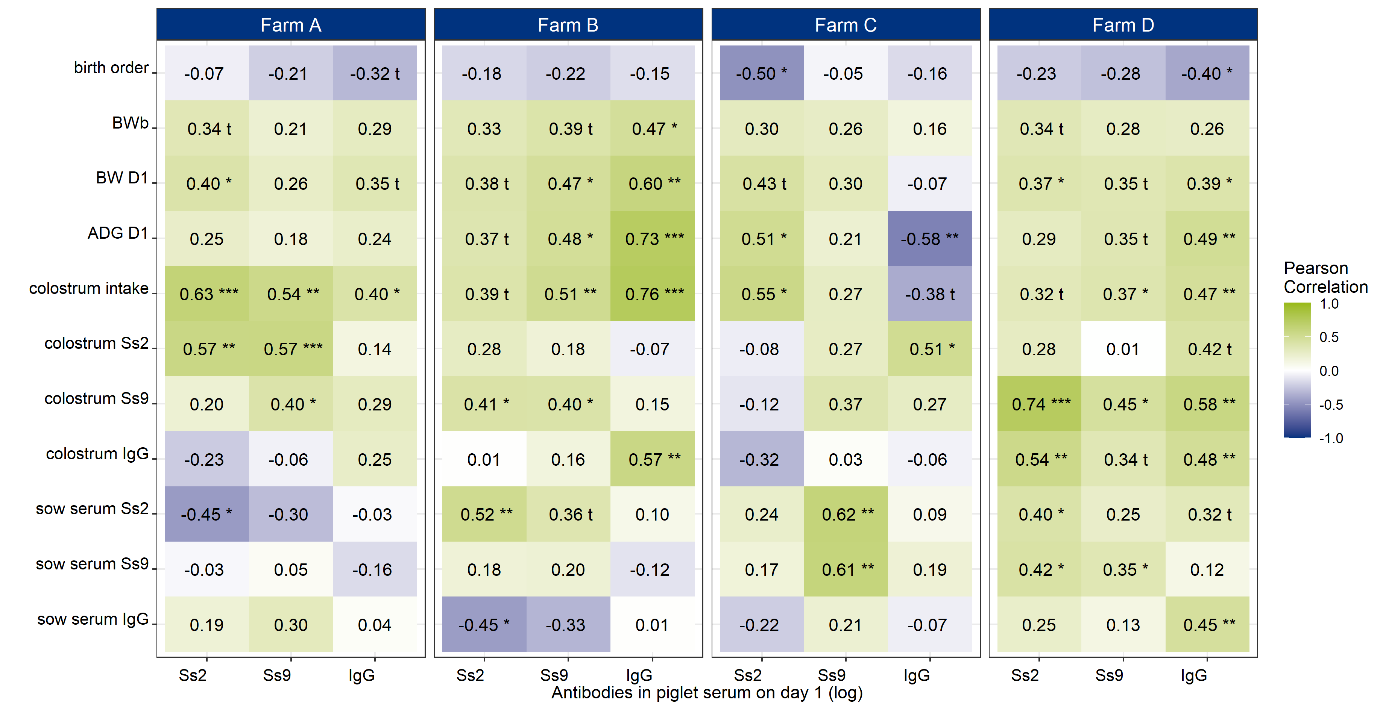


**Figure 2: Correlation between sow and piglet parameters around birth for total *S. suis* specific (serotype 2 and 9) and total antibody levels in piglets serum per farm.** **(A-D)** The Pearson’s correlation coefficient per farm (A-D) was calculated. A one sample T-test was performed to determine which correlation coefficients were significantly different from zero; Significant differences are indicated by asterisks, with , t < 0.1, * P < 0.05, ** P < 0.01, *** P < 0.001. Blue indicates a negative correlation and green indicates a positive correlation. The parameters used to determine the association as described above were: body weight birth (BWb), body weight day one (BW D1); average daily day one (ADG D1); *S. Suis* reacting antibodies serotype 2 (Ss2) and serotype (9); colostrum intake estimated on calculation Theil (1).

**Figure 3 Kinetics *S. suis* specific antibodies in pigs from day 1 till day 69 per litter.** Serum samples from six piglets per litter (ten litters in total) were analyzed by ELISA and mean results and SD are shown; **(A )** The level of *S. suis* serotype 2 reacting antibodies (total Ig) **(B)** The level of serotype 9 reacting antibodies (total Ig) expressed relative to a positive control serum (% positivity).


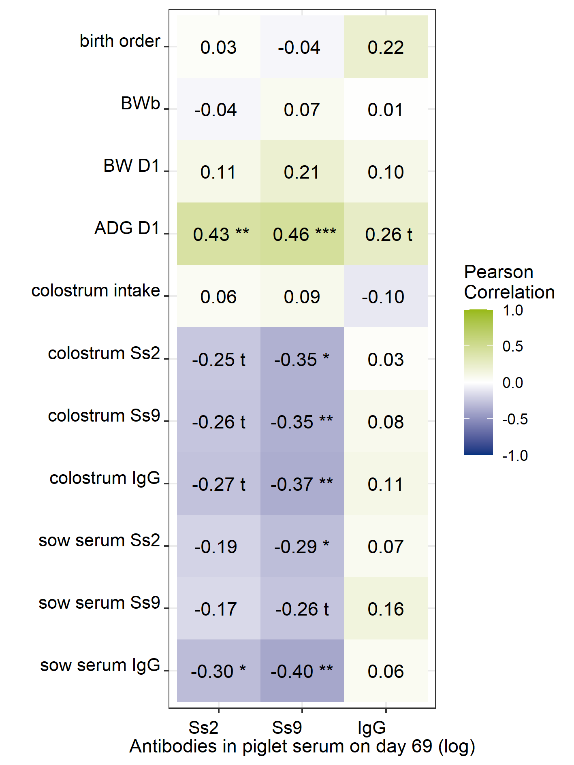

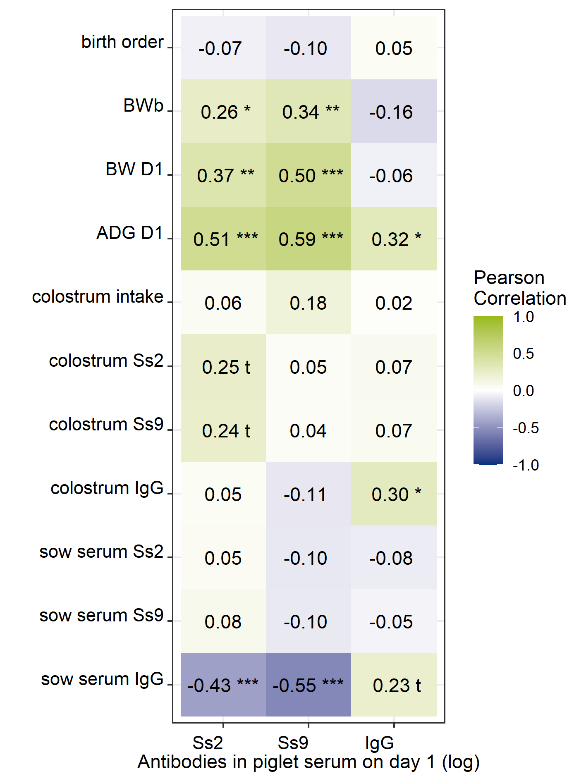


**Figure 4 Correlation between sow and piglet parameters for total *S. suis* specific (serotype 2 and 9) and total antibody levels in piglets serum for farm B during the kinetic study. (A)** on day one; **(B)** on day 69; The Pearson’s correlation coefficient was calculated; Significant differences are indicated by asterisks, with , t < 0.1, * P < 0.05, ** P < 0.01, *** P < 0.001. Blue indicates a negative correlation and green indicates a positive correlation; The parameters used to determine the associations as described above were: body weight birth (BWb), body weight day one (BW D1); average daily day one (ADG D1); *S. suis reacting antibodies* serotype 2 (Ss2) and serotype (9); colostrum intake estimated on calculation Theil (1).

1. Theil PK, Flummer C, Hurley WL, Kristensen NB, Labouriau RL, Sørensen MT. Mechanistic model to predict colostrum intake based on deuterium oxide dilution technique data and impact of gestation and prefarrowing diets on piglet intake and sow yield of colostrum1. J Anim Sci. 2014;92(12):5507-19.

**Figure 5**

| **Supplementary Figure 5A: the effect of ADG D1 on antibody dynamics** | |
| --- | --- |
| 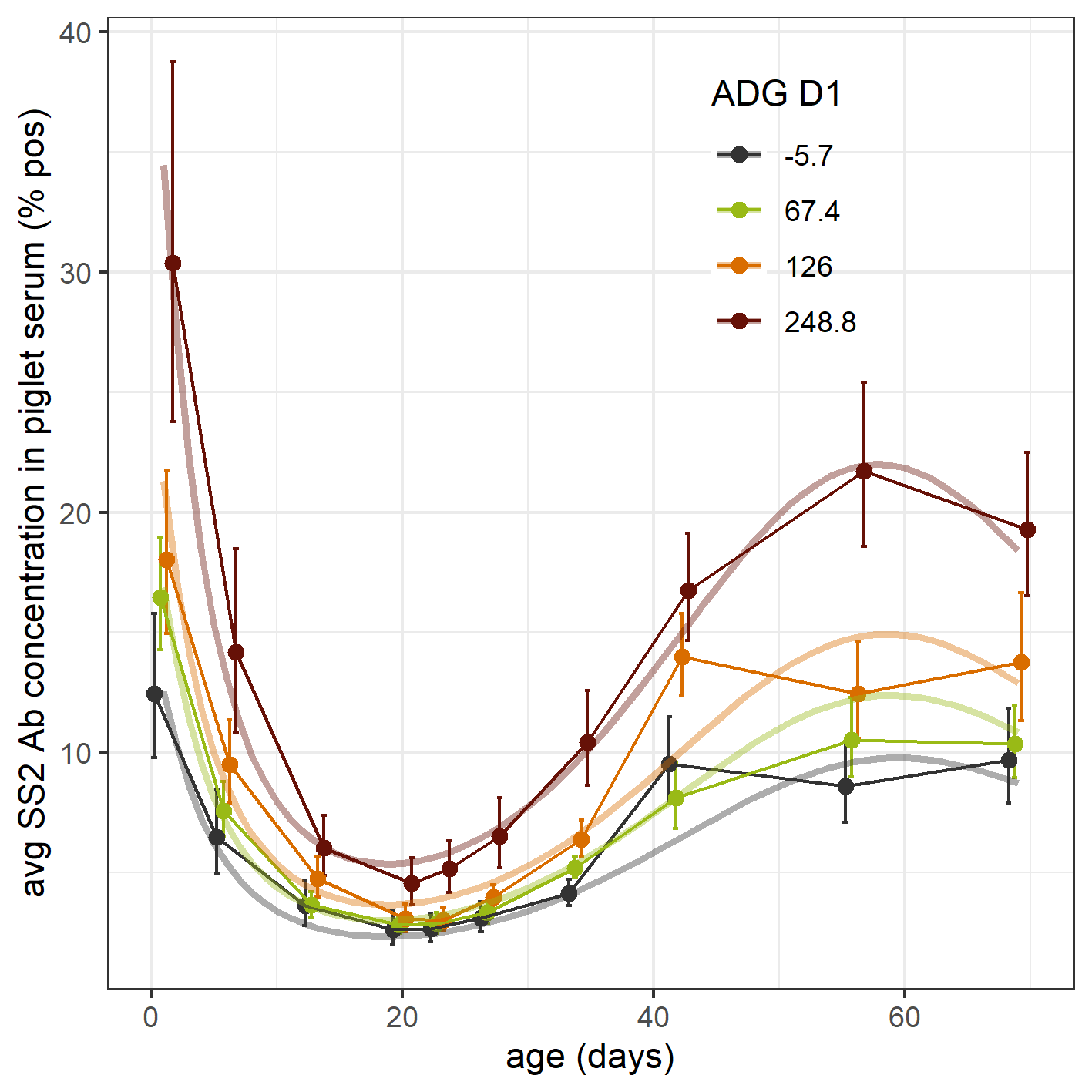 | 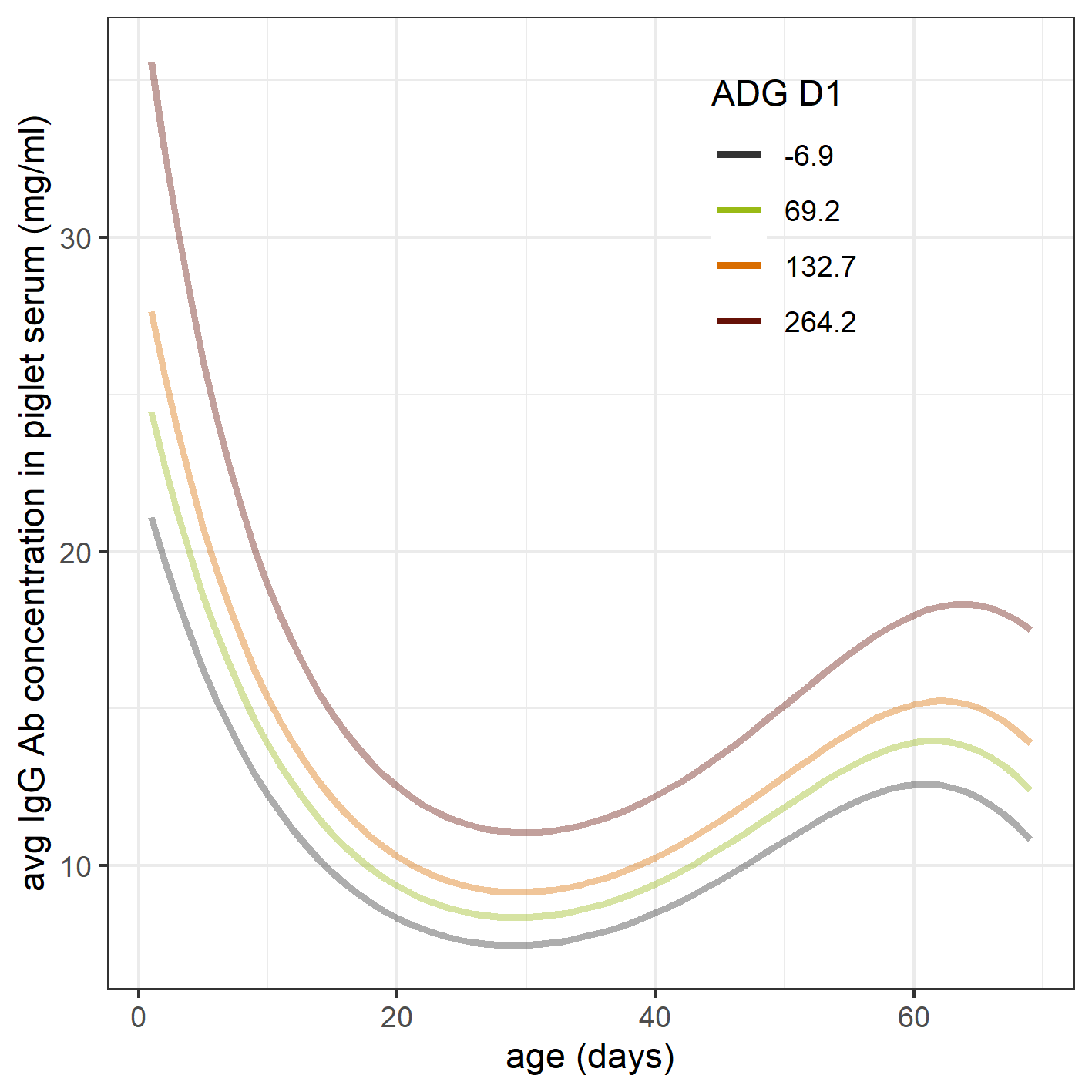 |
| 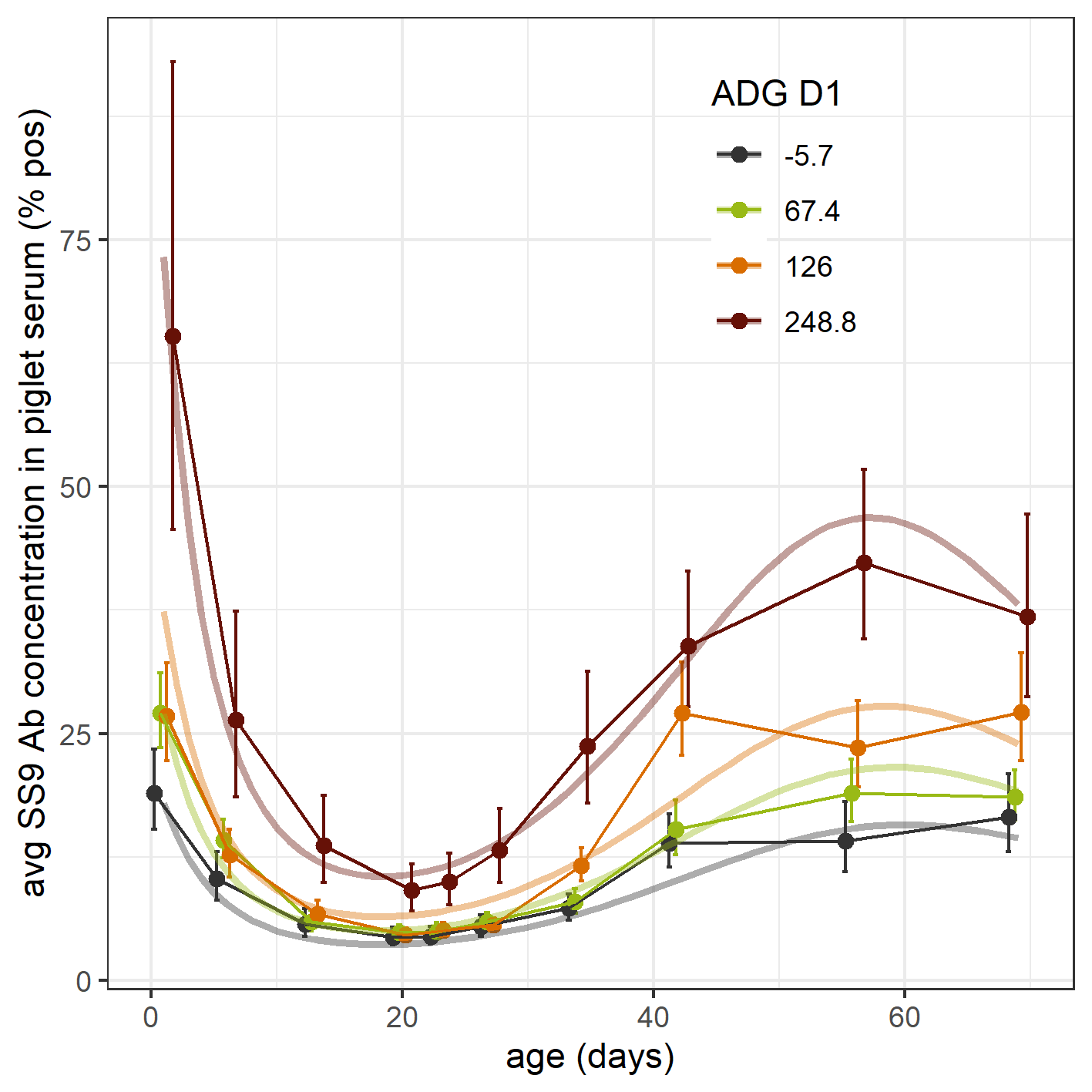 | 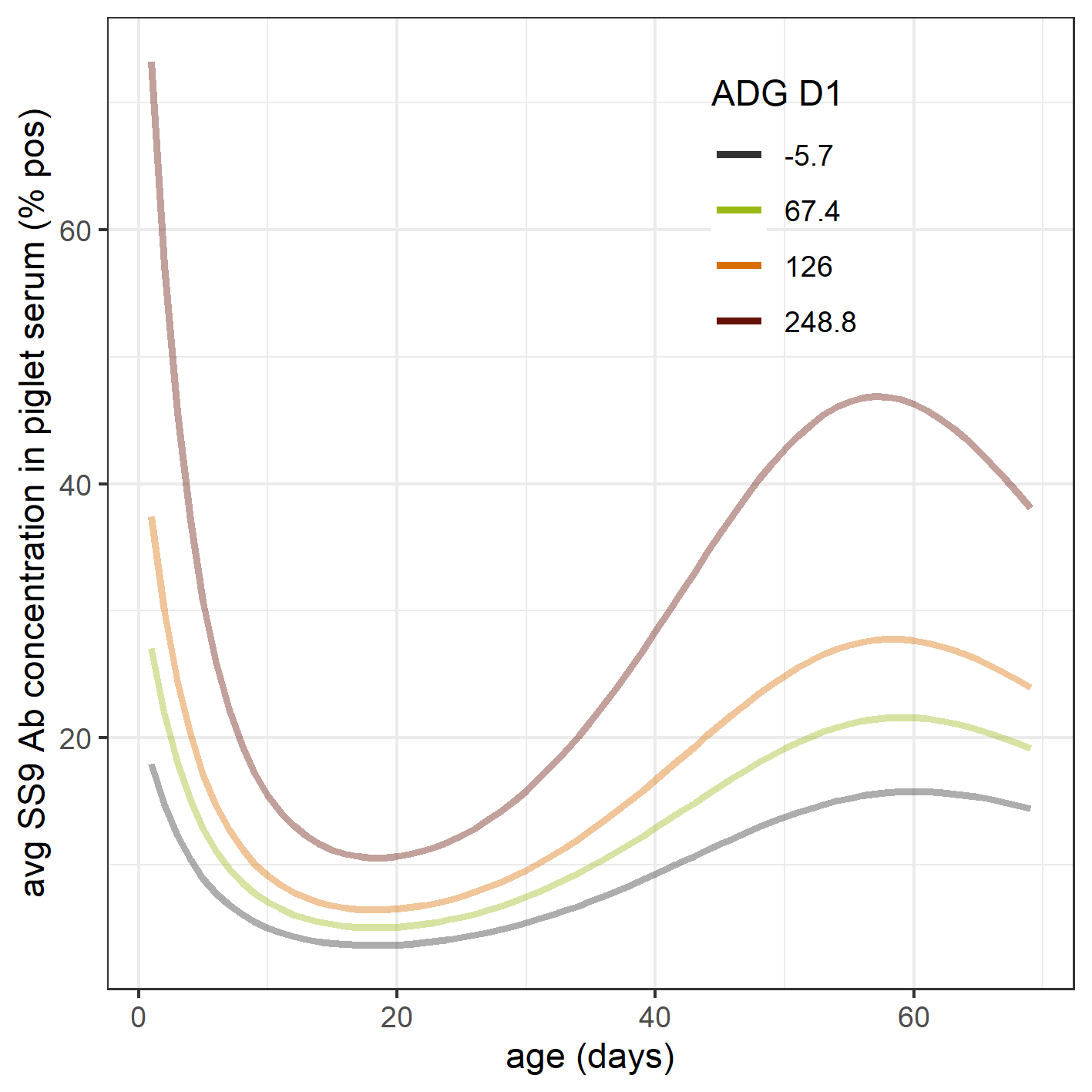 |

**A**: **average daily gain 24-h after birth (ADG D1)-** The left graphic depicts four groups with different ADG D1 levels and relates these levels to the % positive (pos) for *S. suis* specific antibodies for serotype 2 (Ss2, first row) and serotype 9 (Ss9, second row). For all ten blood sampling time-points the standard error of each group is shown. The right graphic depicts only the modelled version.

| **Supplementary Figure 5B: the effect of ADG 34 on antibody dynamics** | |
| --- | --- |
| 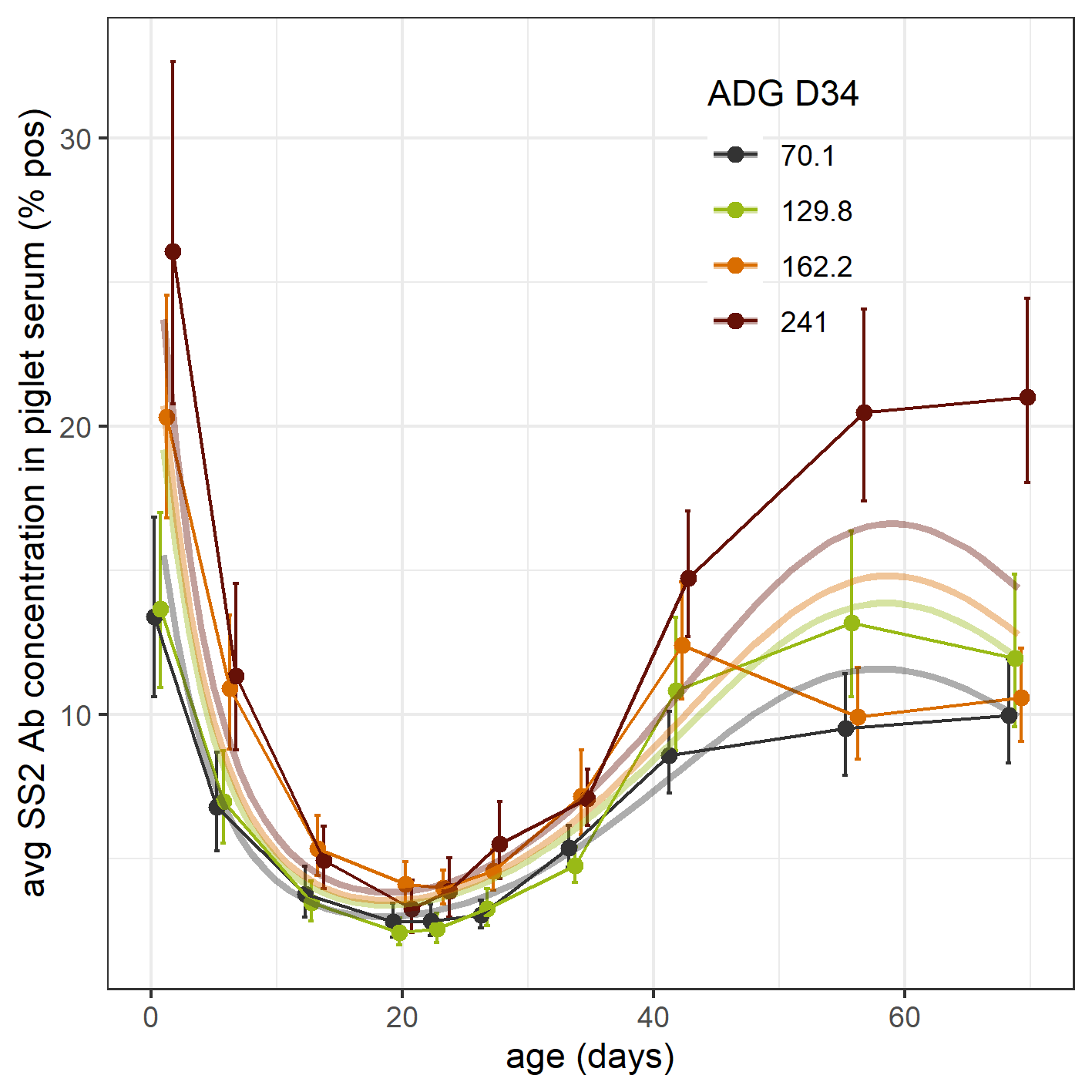 | 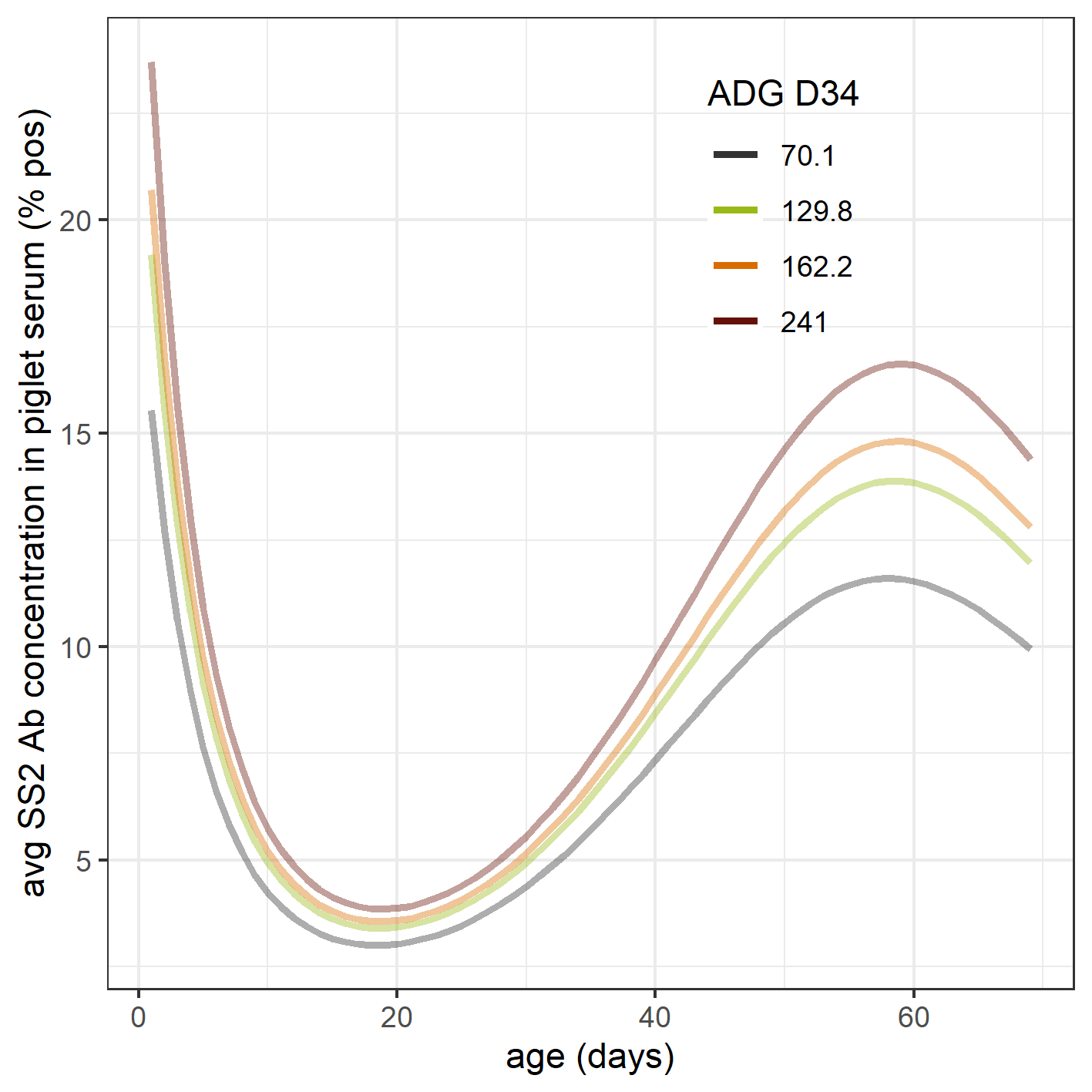 |
| 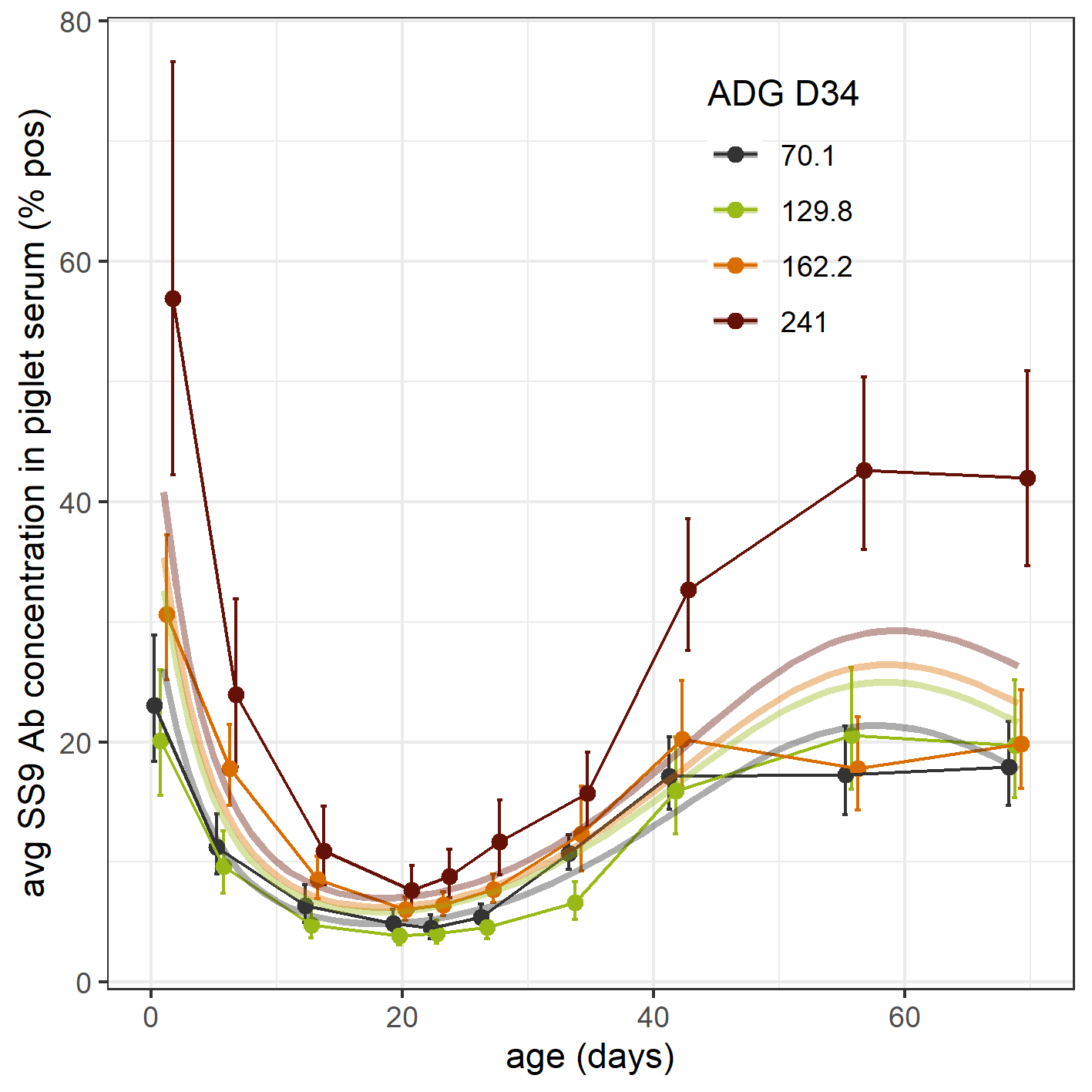 | 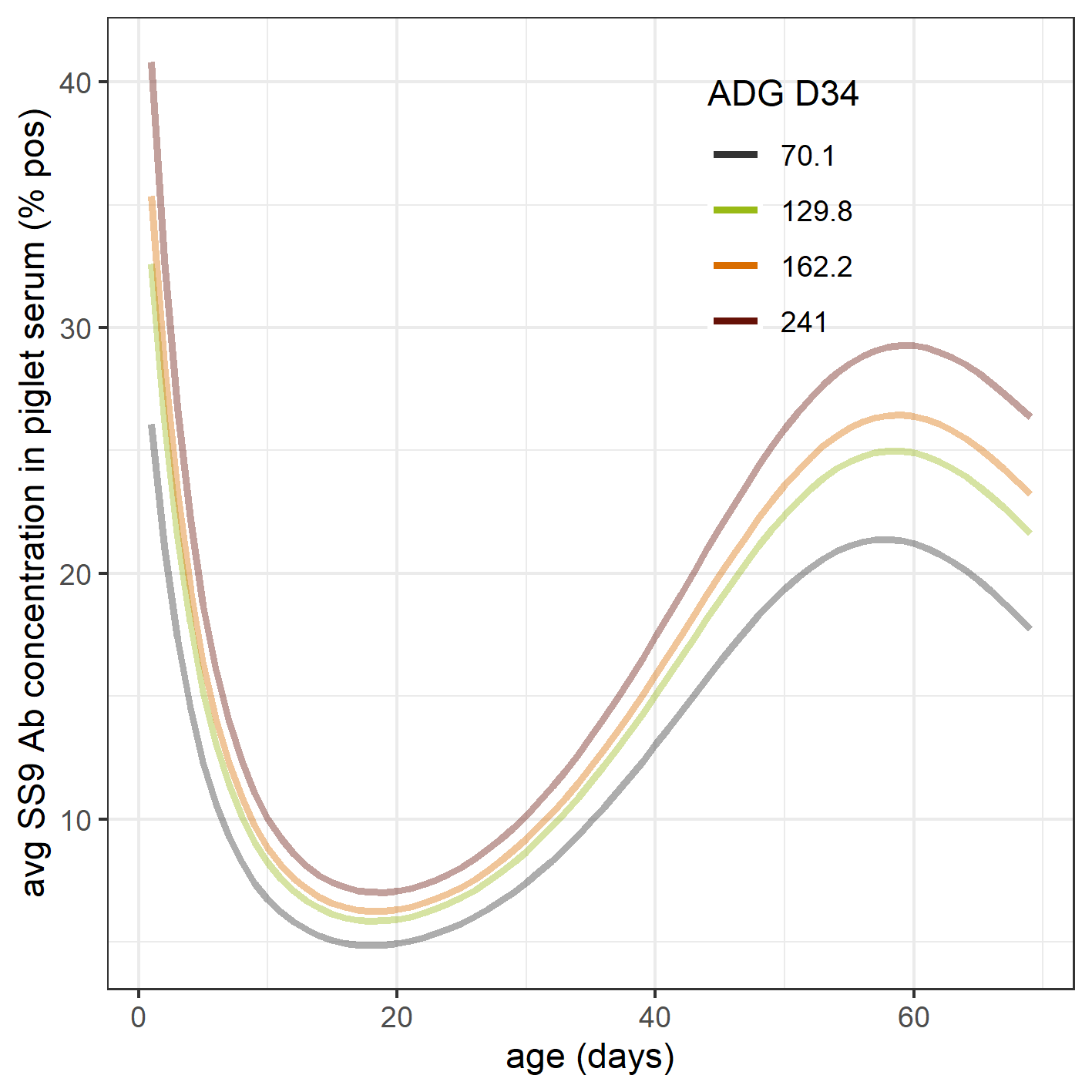 |

**B: average daily gain on day 34 (ADG D34)**- The left graphic depicts four groups with different ADG D34 levels and relates these levels to the % positive (pos) for *S. suis* specific antibodies for serotype 2 (Ss2, first row) and serotype 9 (Ss9, second row). For all ten blood sampling time-points the standard error of each group is shown. The right graphic depicts only the modelled version.

| **Supplementary Figure 5C: the effect of BW D1 on antibody dynamics** | |
| --- | --- |
| 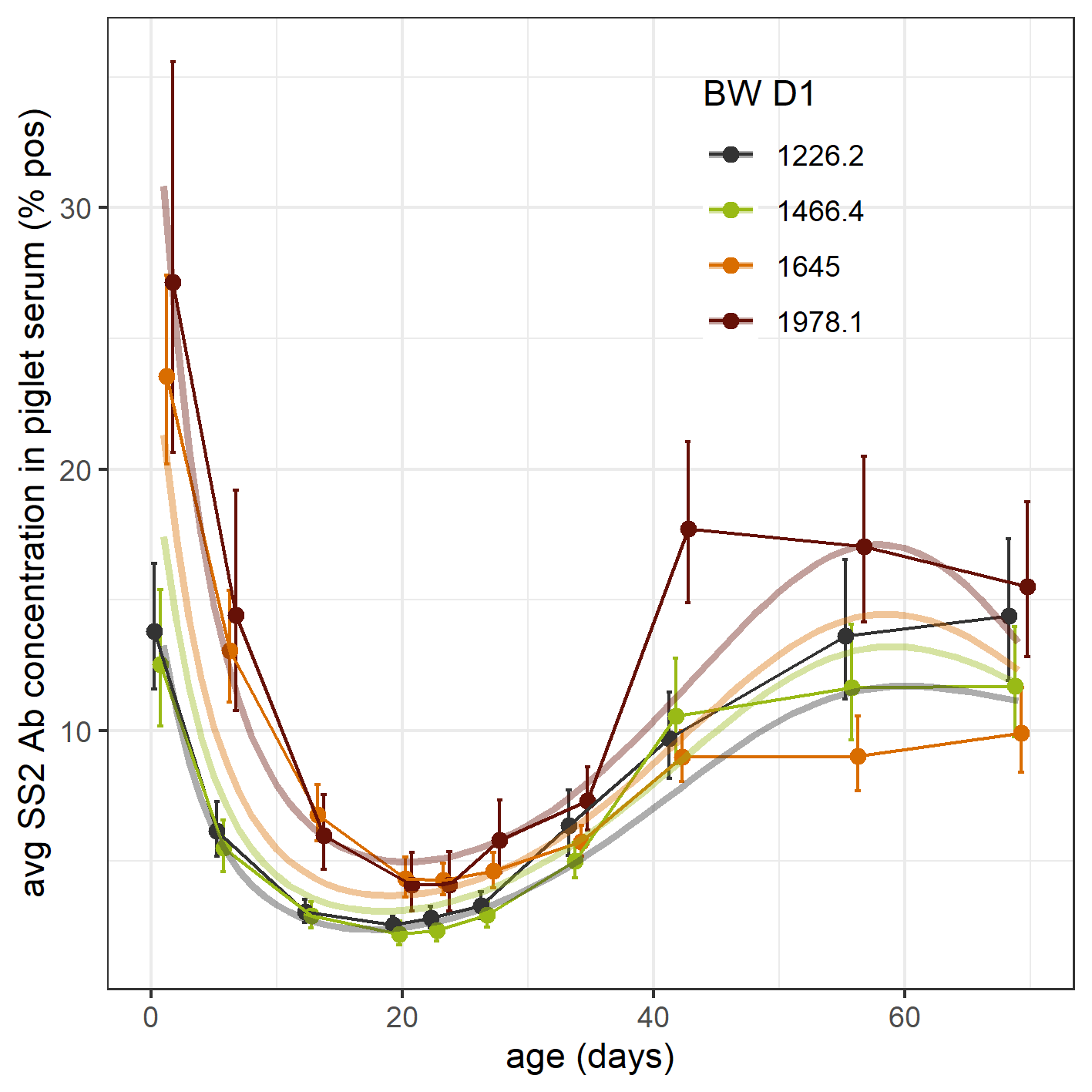 | 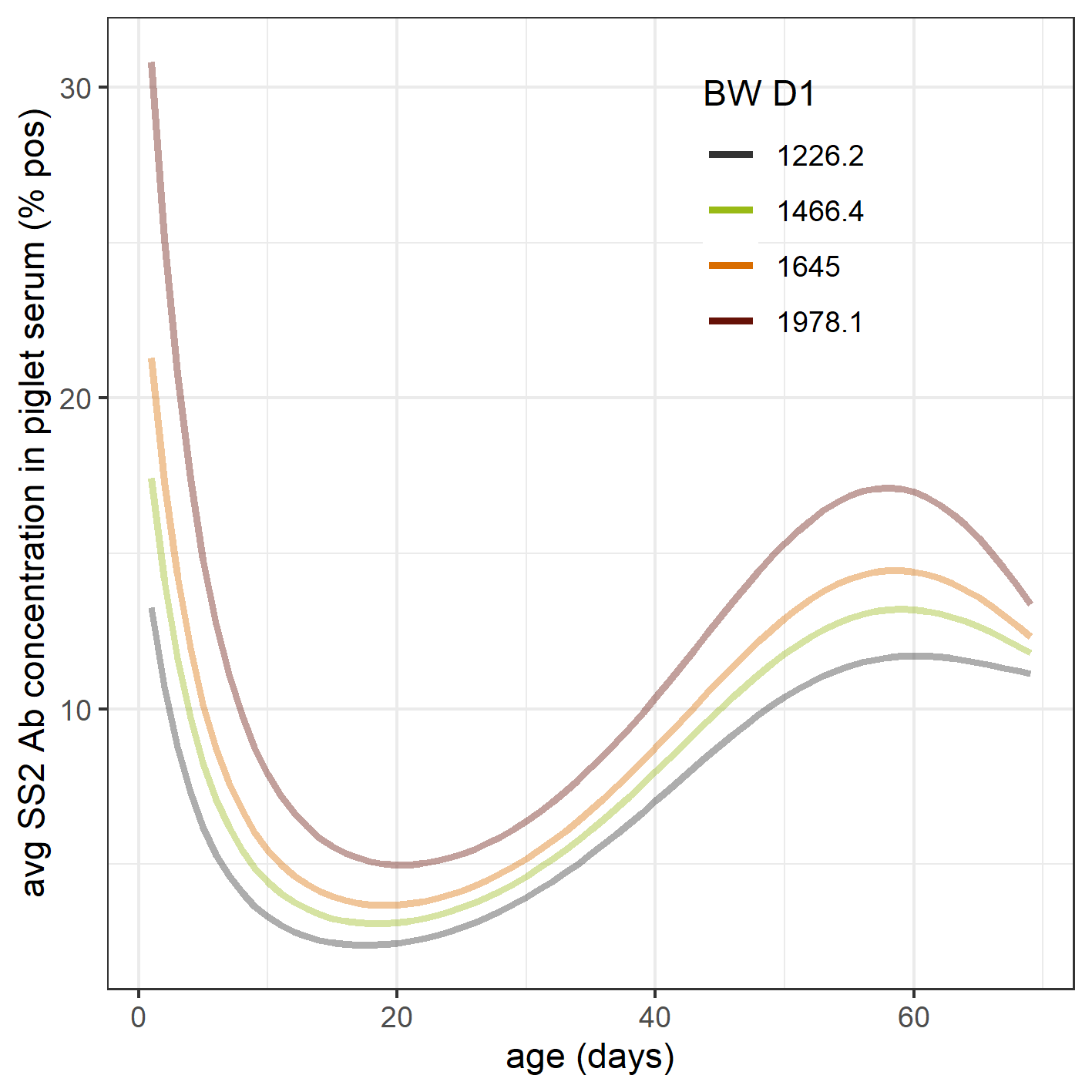 |
| 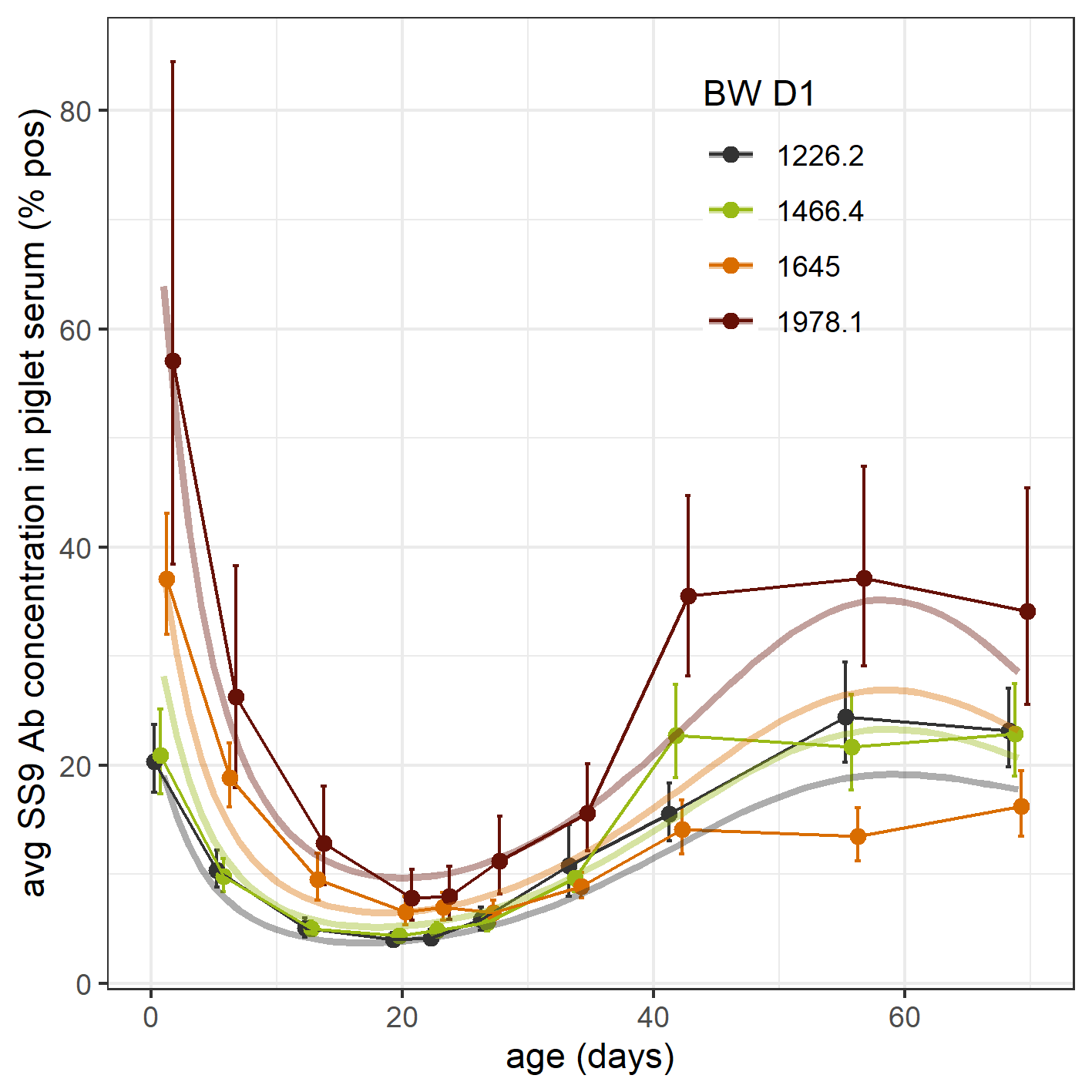 | 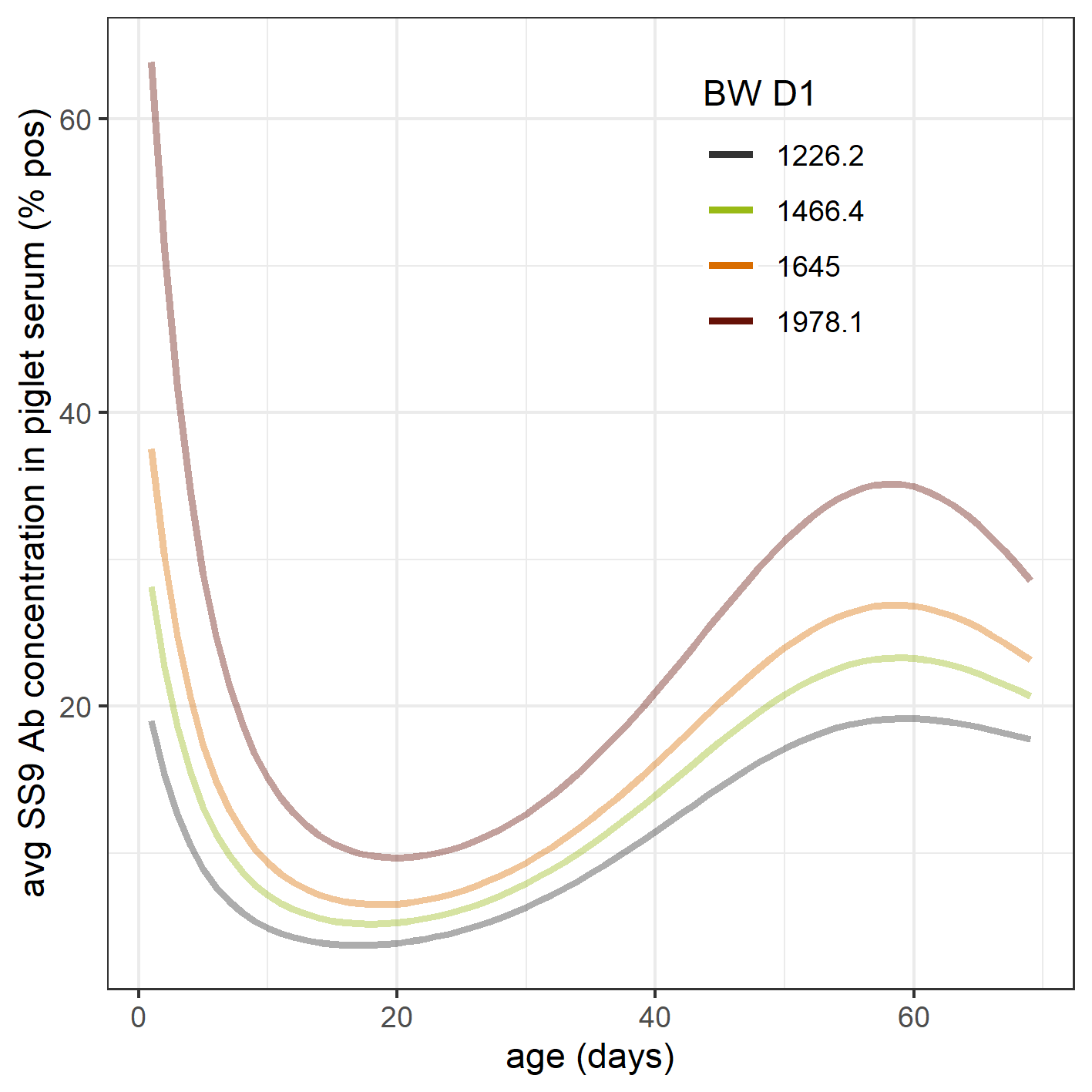 |

**C: Body weight one day after birth (BW D1**) - The left graphic depicts four groups with different BW D1 levels and relates these levels to the % positive (pos) for *S. suis* specific antibodies for serotype 2 (Ss2, first row) and serotype 9 (Ss9, second row). For all ten blood sampling time-points the standard error of each group is shown. The right graphic depicts only the modelled version.

| **Supplementary Figure 5D: the effect of sow colostrum IgG on antibody dynamics** | |
| --- | --- |
| 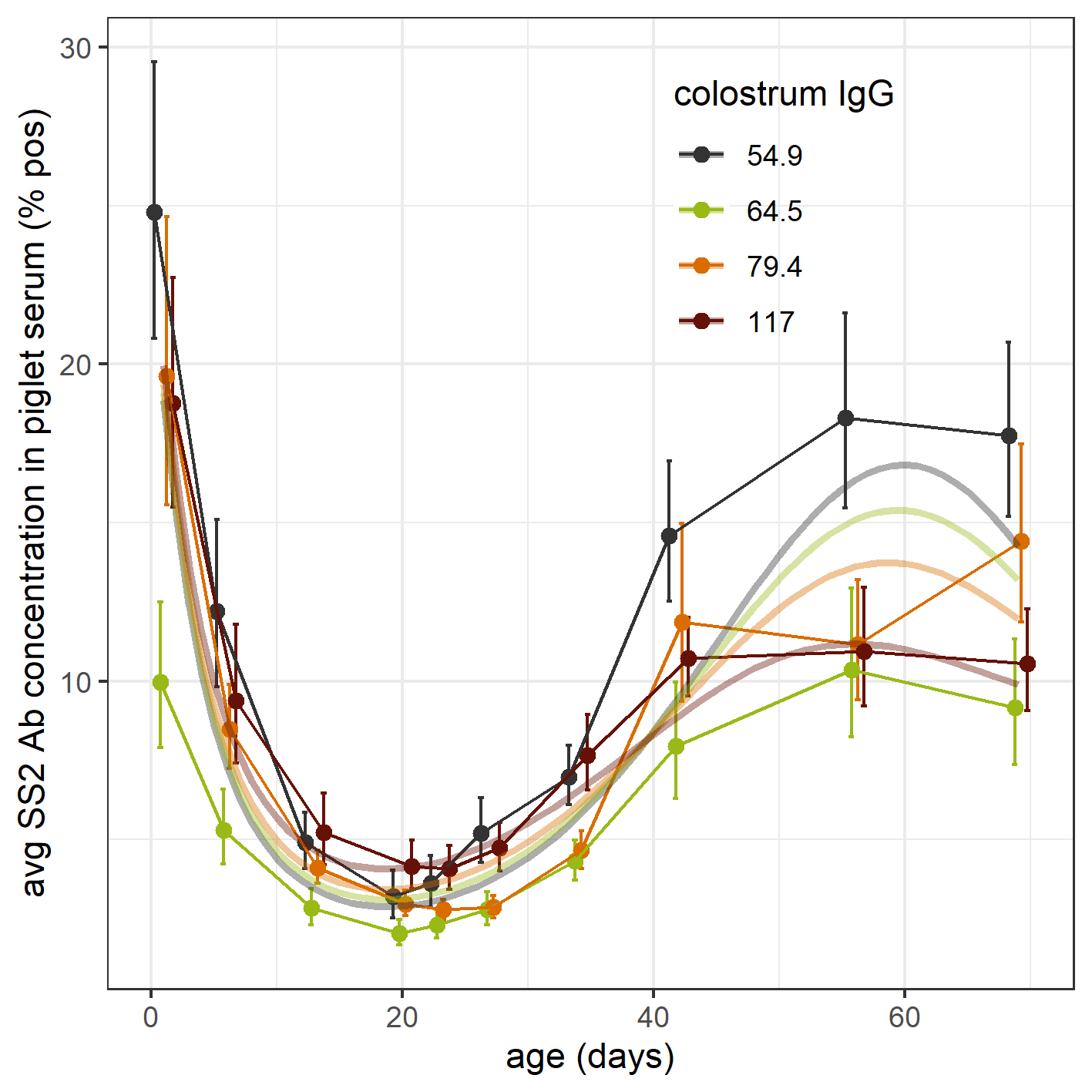 | 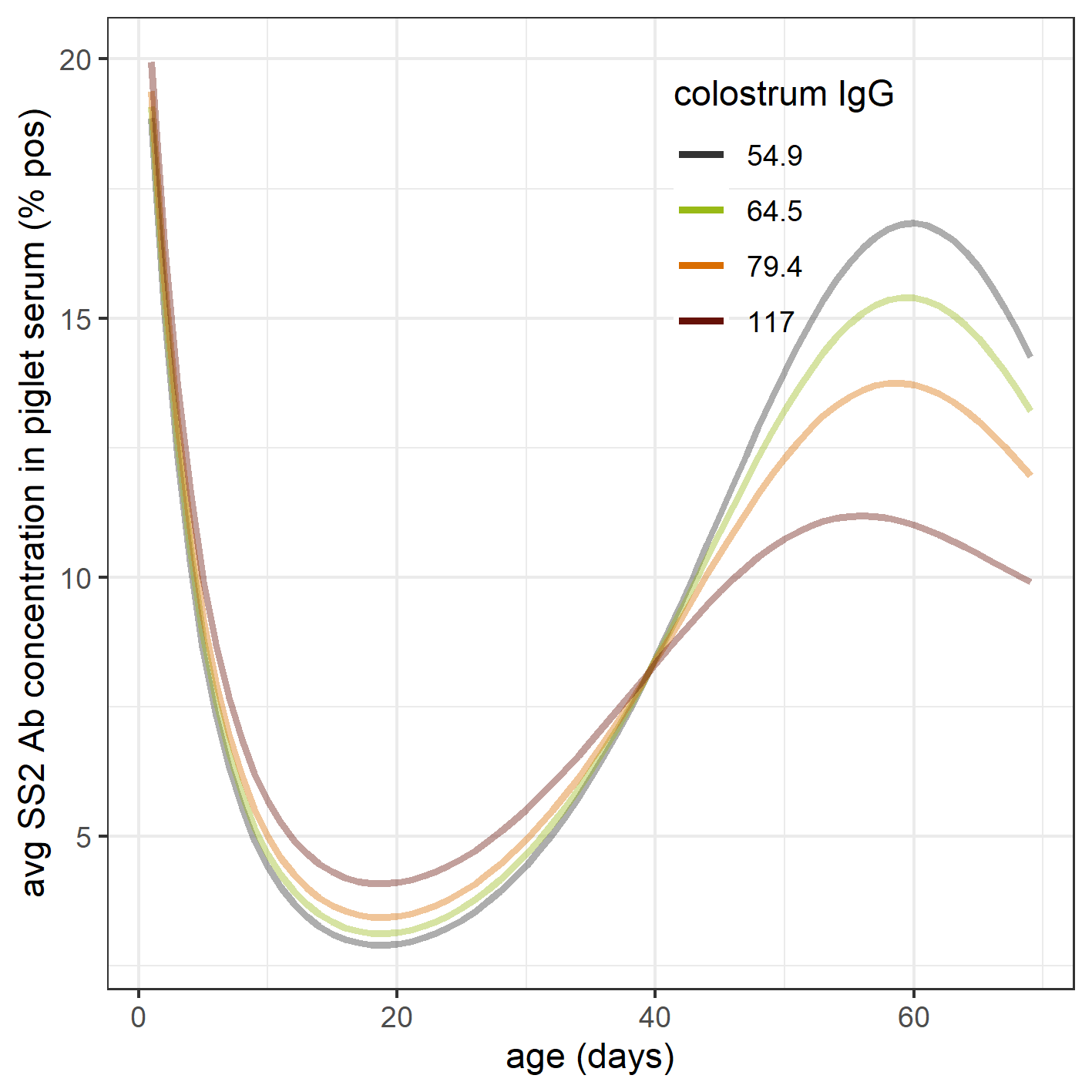 |
| 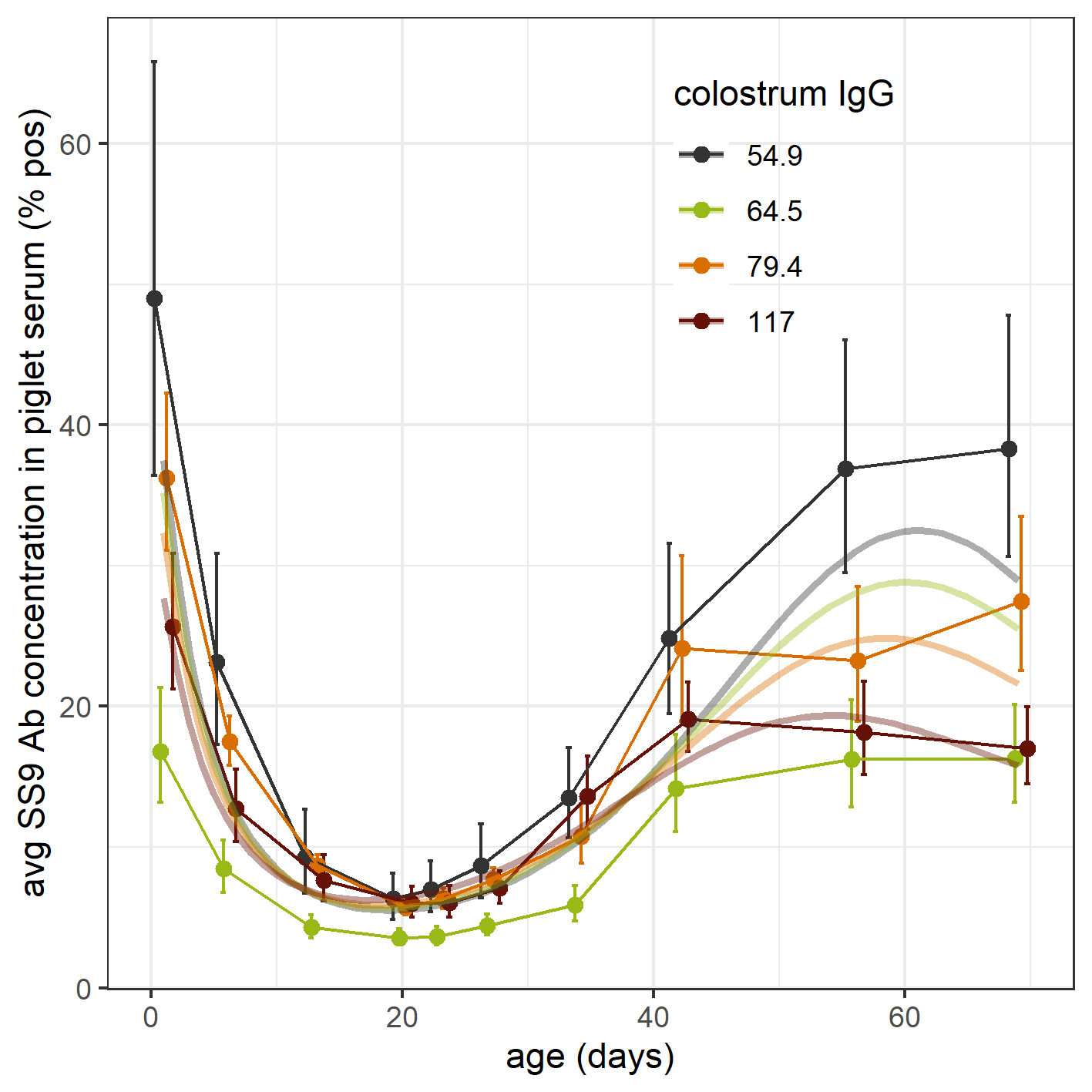 | 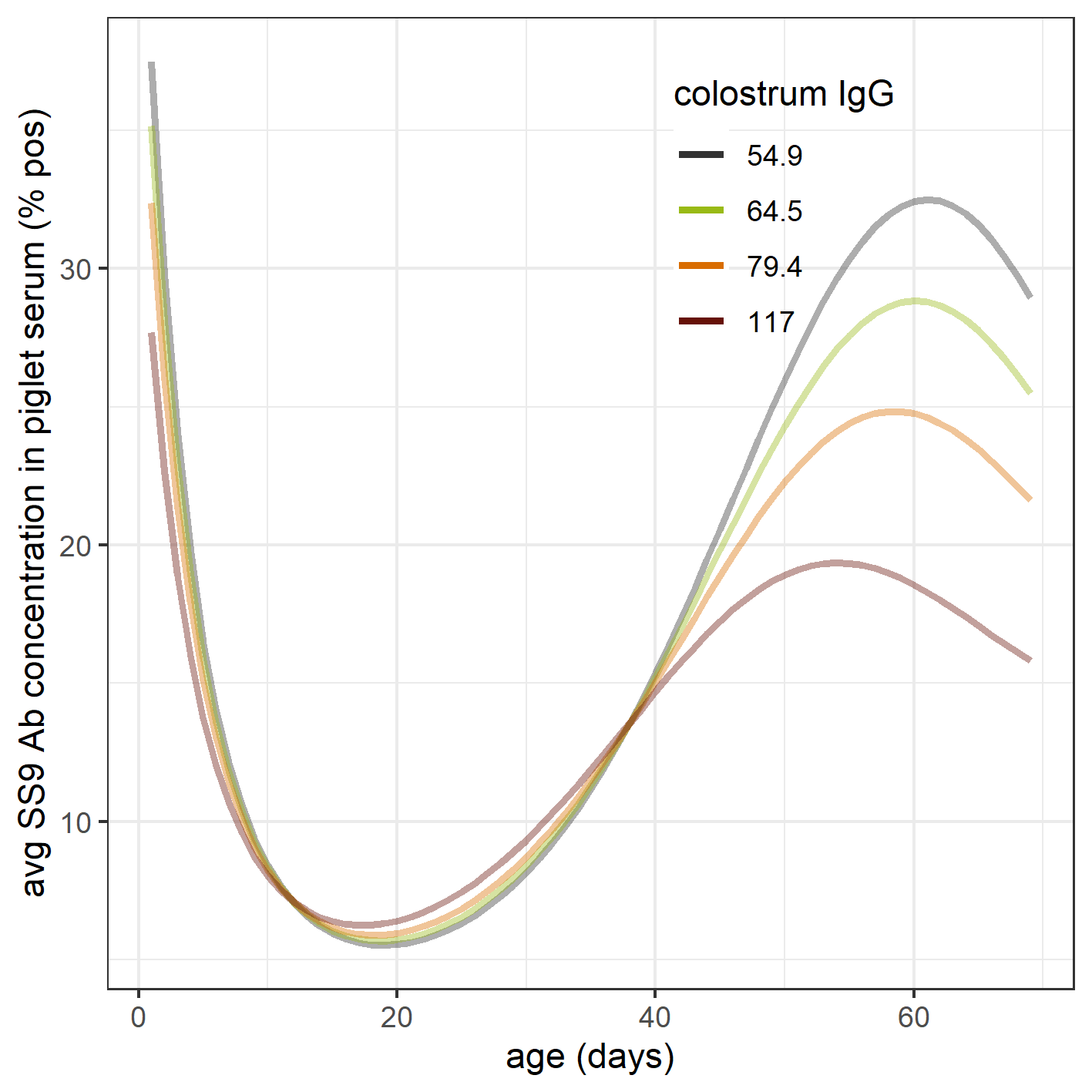 |

**D**: **Sow colostrum IgG** - The left graphic depicts four groups with different sow colostrum IgG levels and relates these levels to the % positive (pos) for *S. suis* specific antibodies for serotype 2 (Ss2, first row) and serotype 9 (Ss9, second row). For all ten blood sampling time-points the standard error of each group is shown. The right graphic depicts only the modelled version.

| **Supplementary Figure 5E: the effect of sow colostrum Ss2 on antibody dynamics** | |
| --- | --- |
| 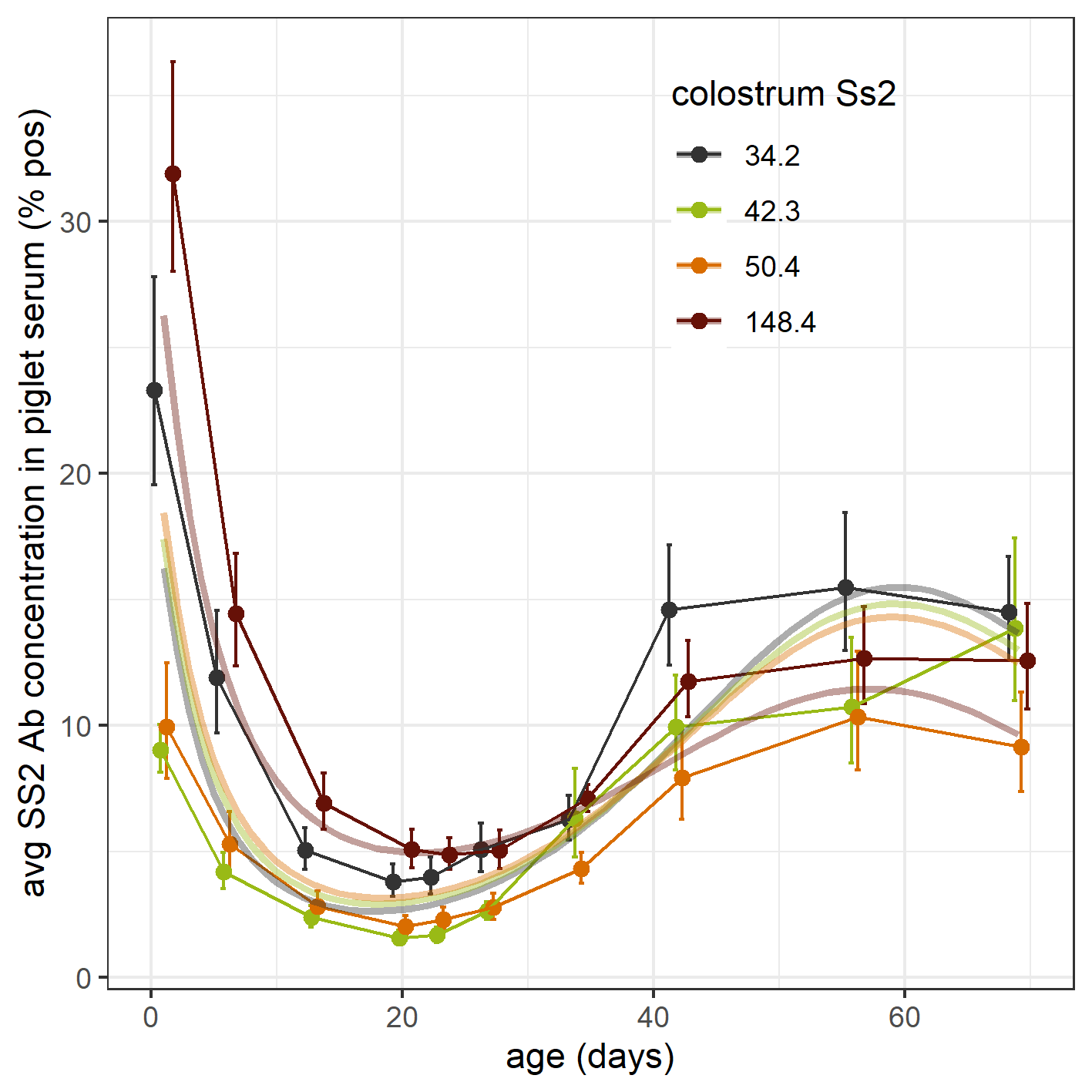 | 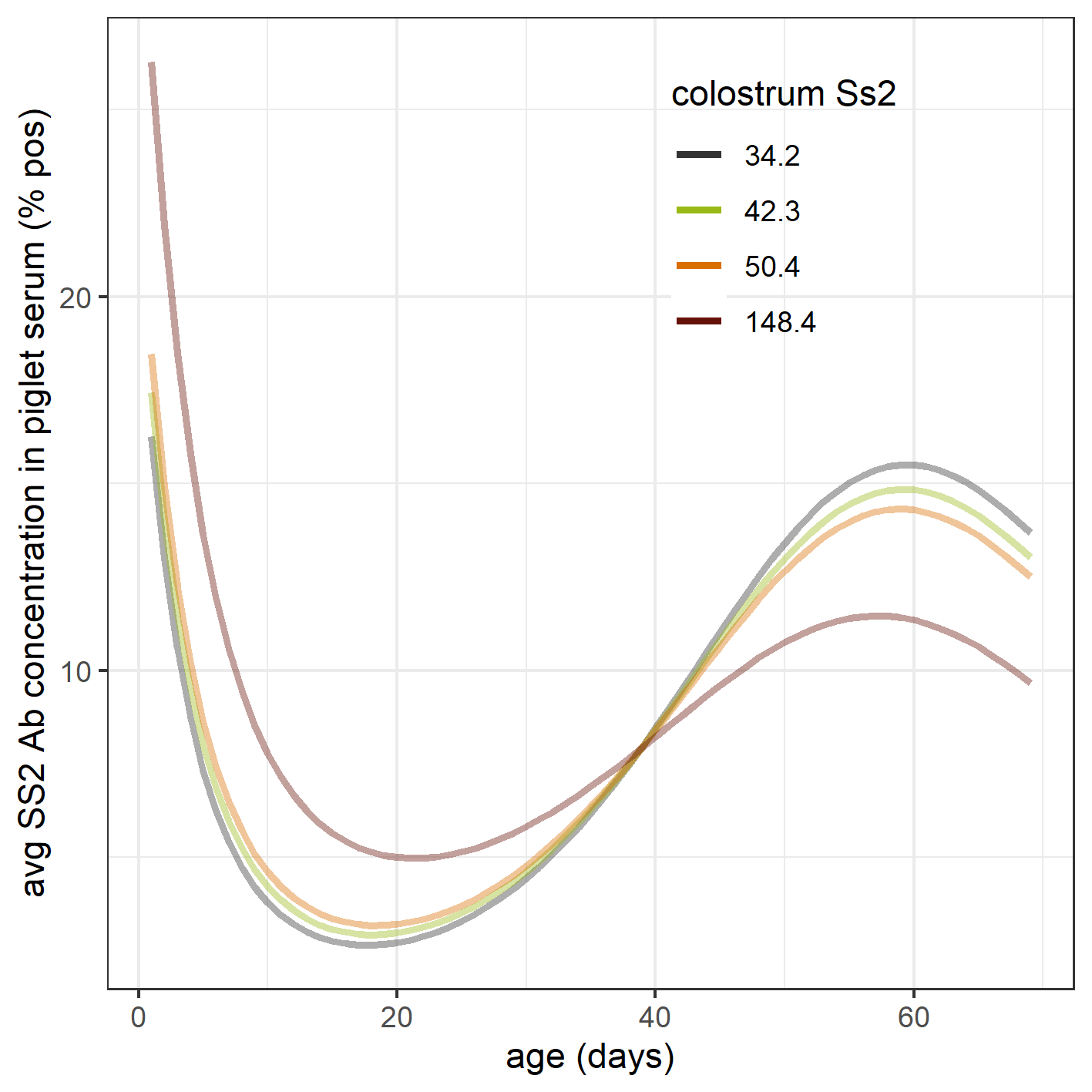 |
| 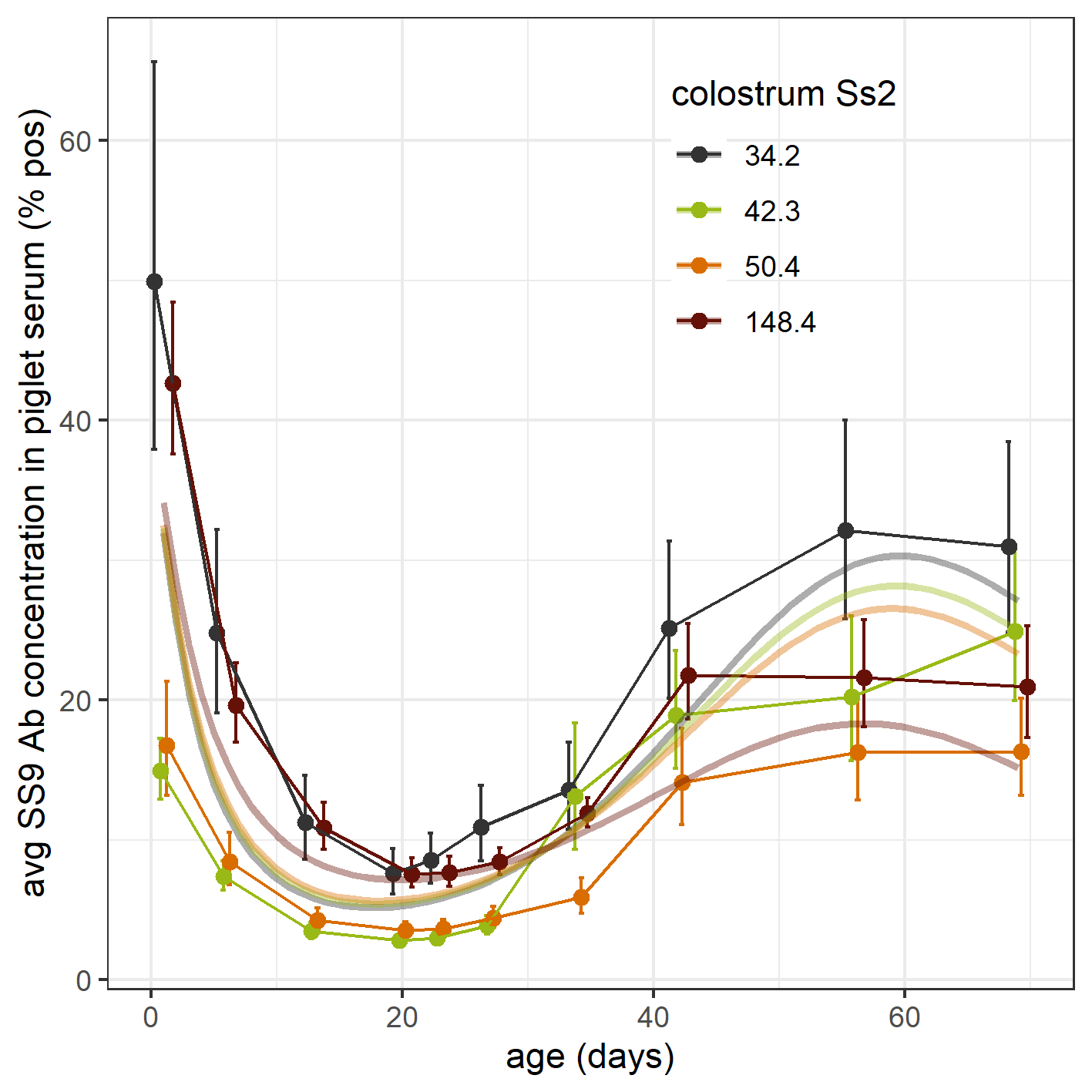 | 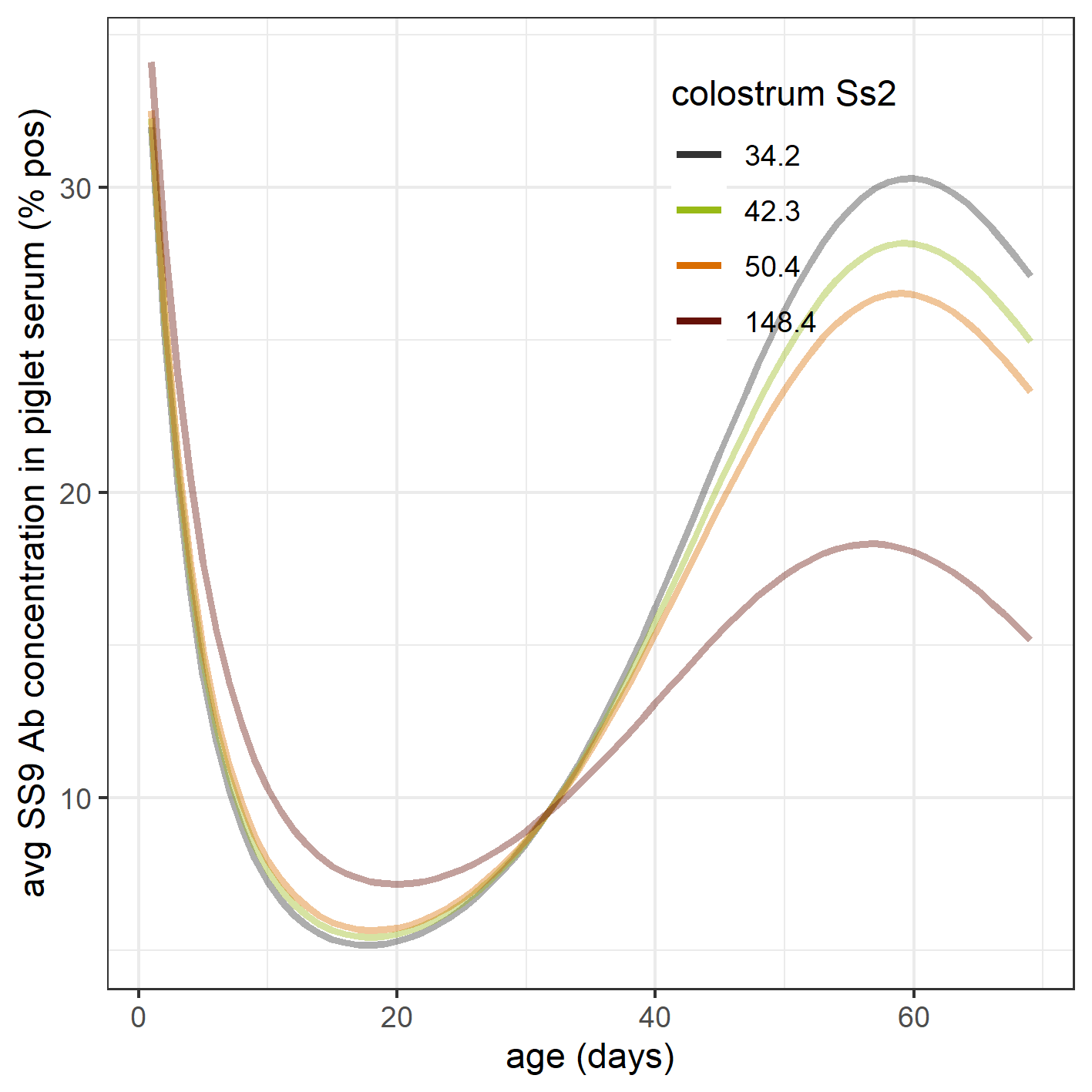 |

**E**: **Sow colostrum Ss2** - The left graphic depicts four groups with different sow colostrum Ss2 levels and relates these levels to the % positive (pos) for *S. suis* specific antibodies for serotype 2 (Ss2, first row) and serotype 9 (Ss9, second row). For all ten blood sampling time-points the standard error of each group is shown. The right graphic depicts only the modelled version.

| **Supplementary Figure 5F: the effect of sow colostrum Ss9 on antibody dynamics** | |
| --- | --- |
| 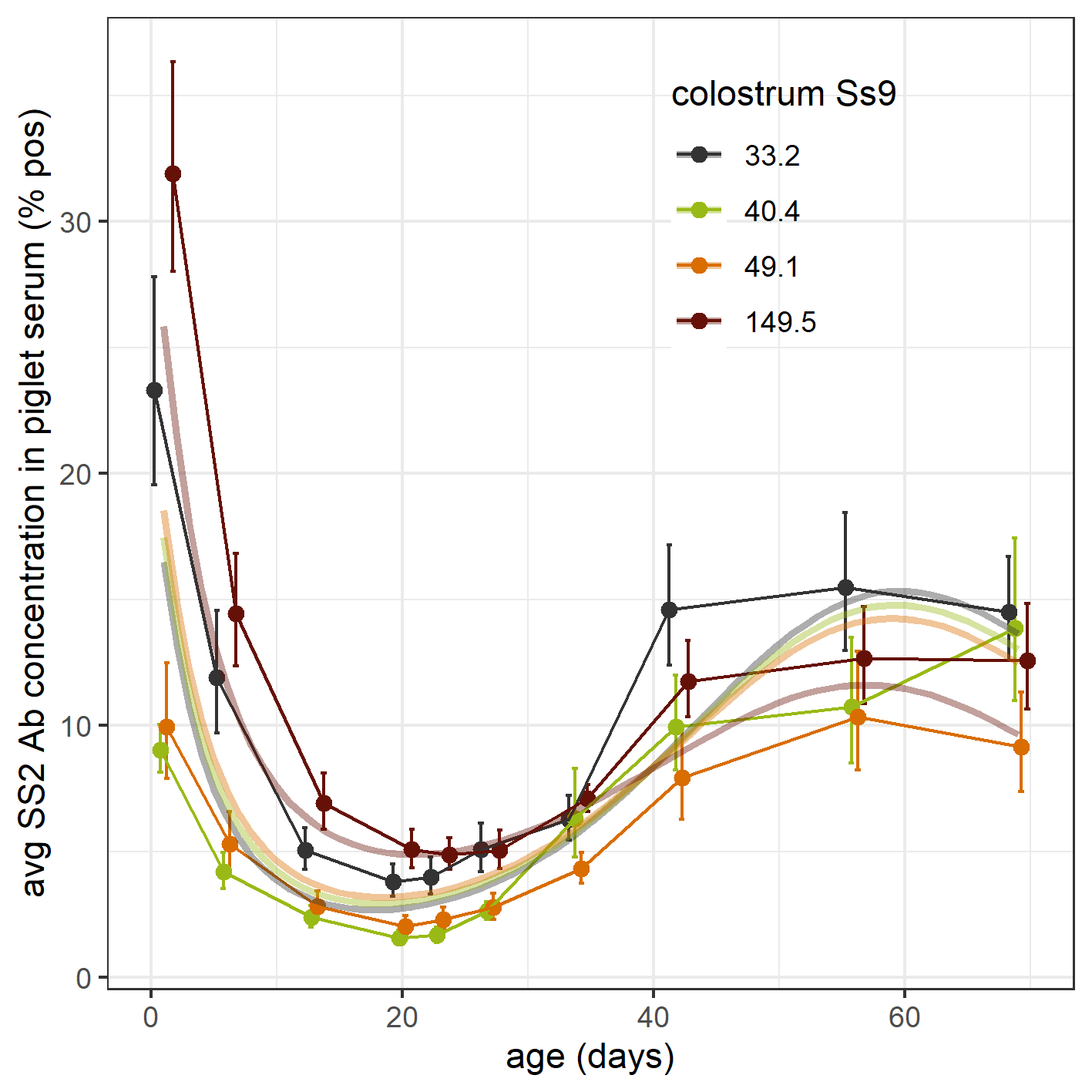 | 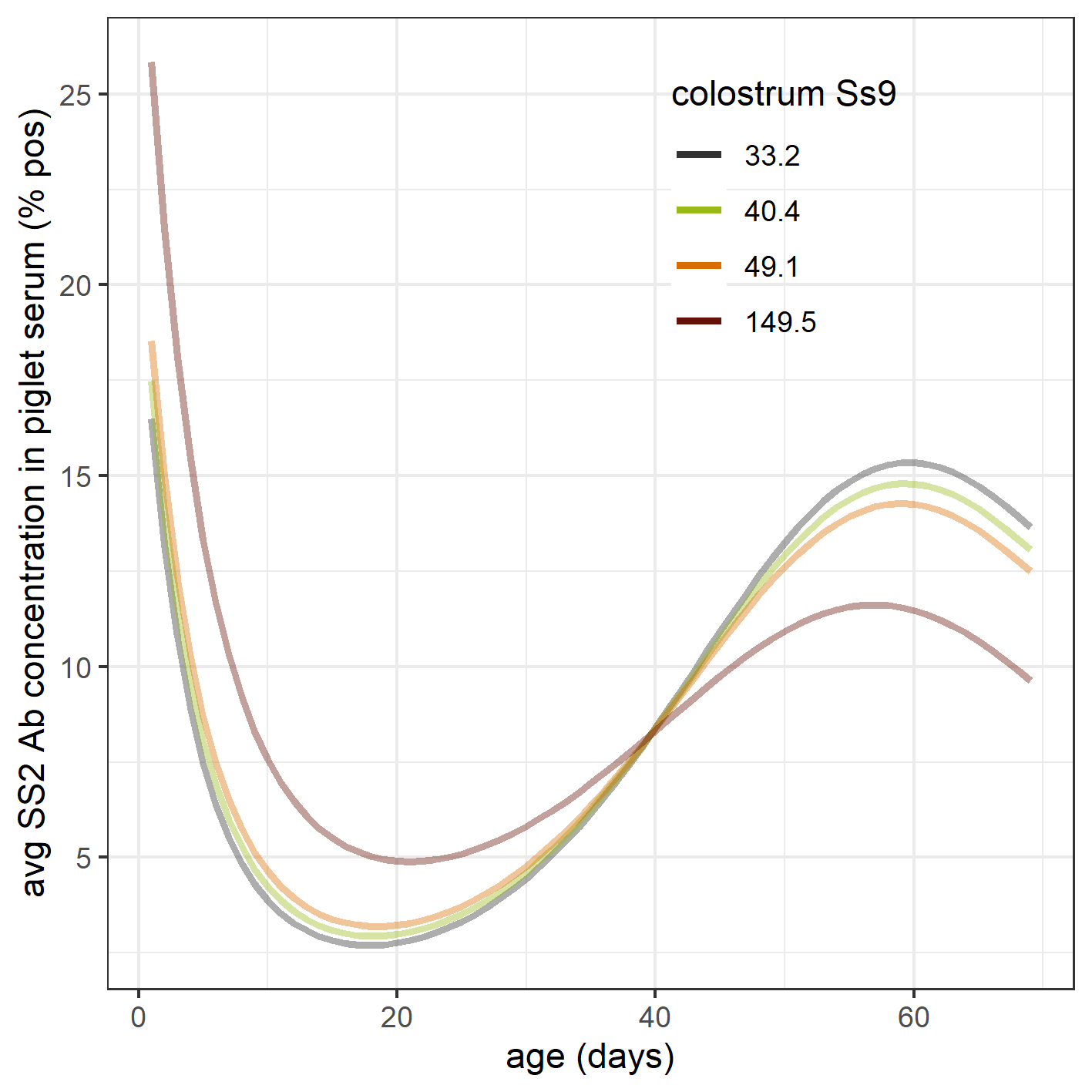 |
| 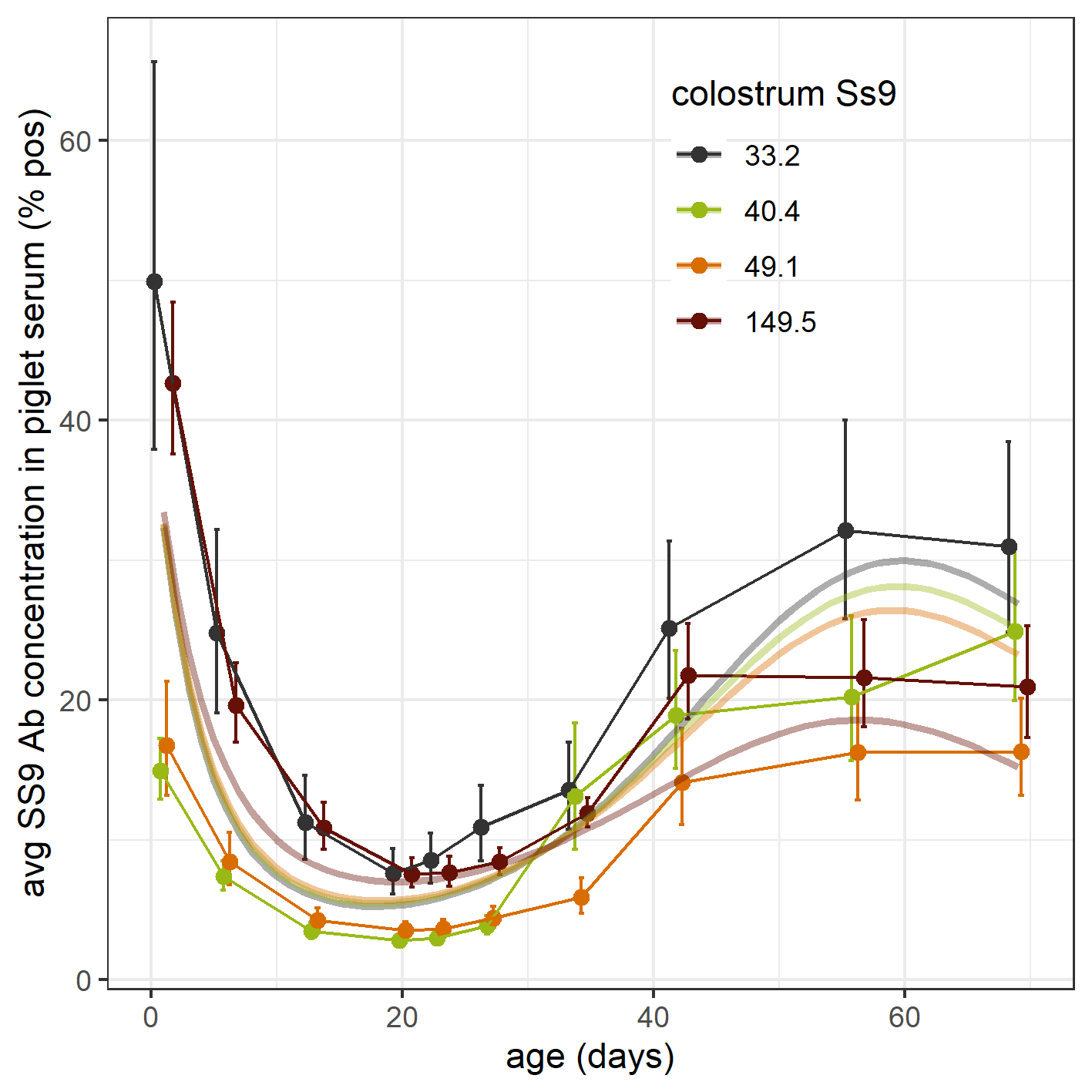 | 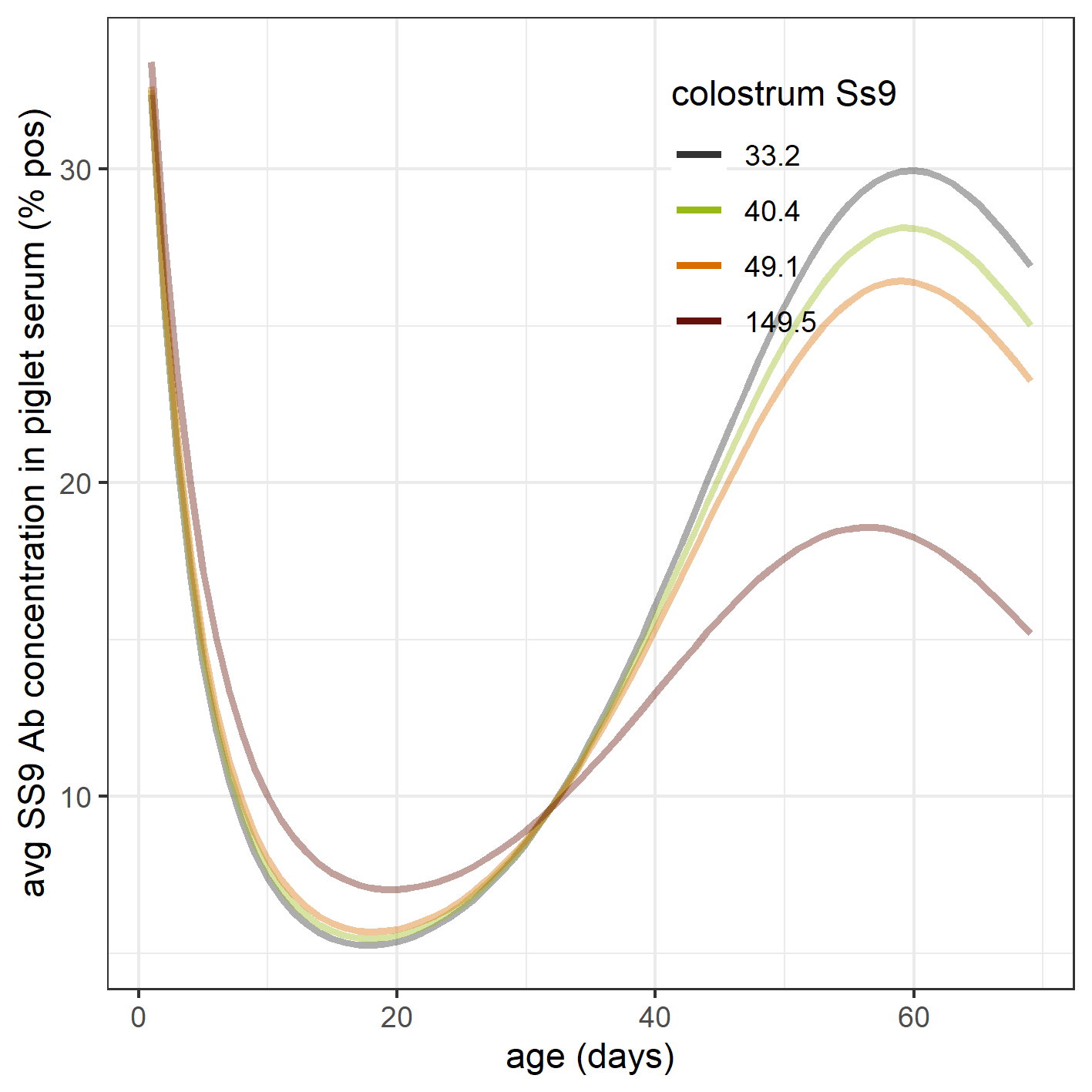 |

**F**: **Sow colostrum Ss9** - The left graphic depicts four groups with different sow colostrum Ss9 levels and relates these levels to the % positive (pos) for *S. suis* specific antibodies for serotype 2 (Ss2, first row) and serotype 9 (Ss9, second row). For all ten blood sampling time-points the standard error of each group is shown. The right graphic depicts only the modelled version.

| **Supplementary Figure 5G: the effect of sow serum IgG on antibody dynamics** | |
| --- | --- |
| 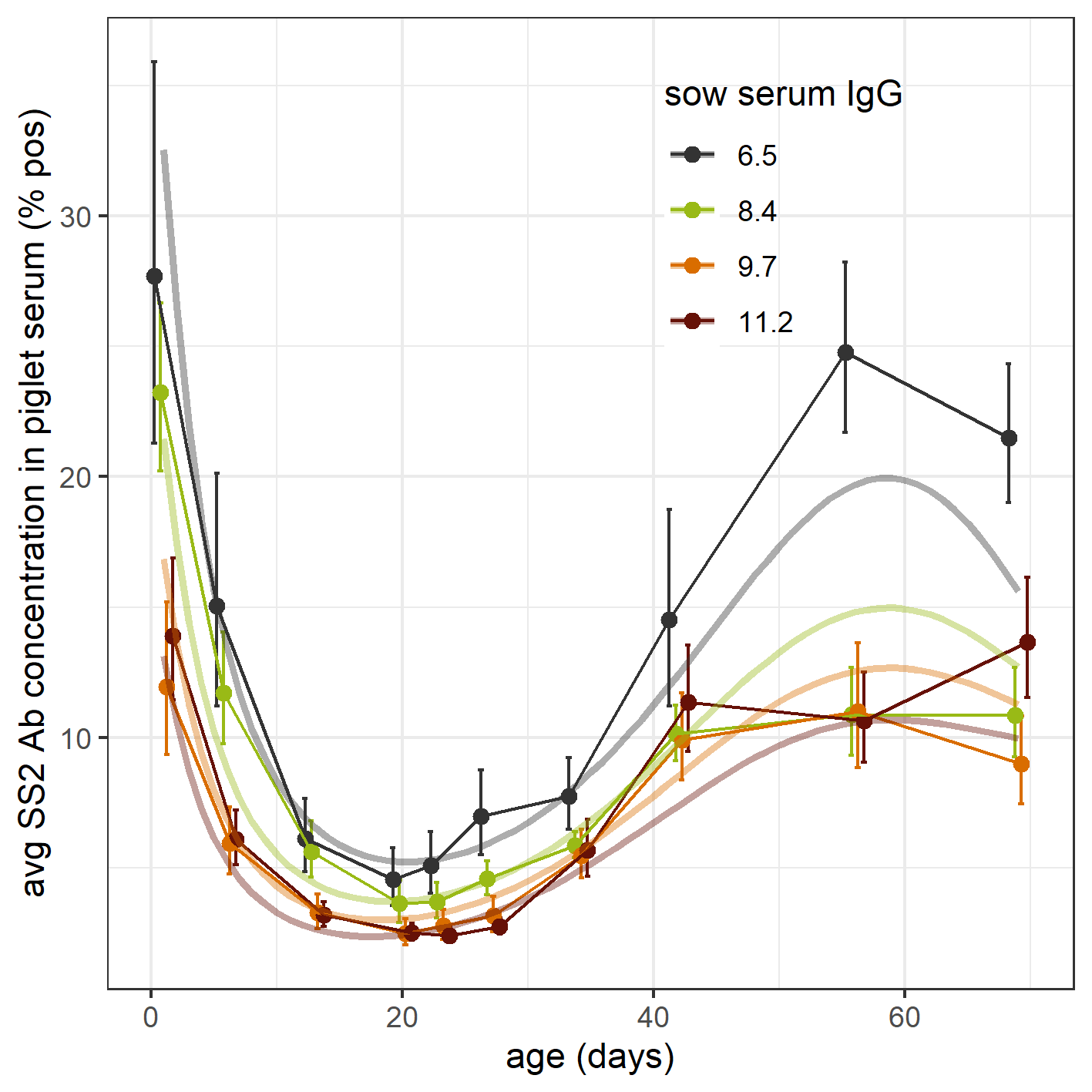 | 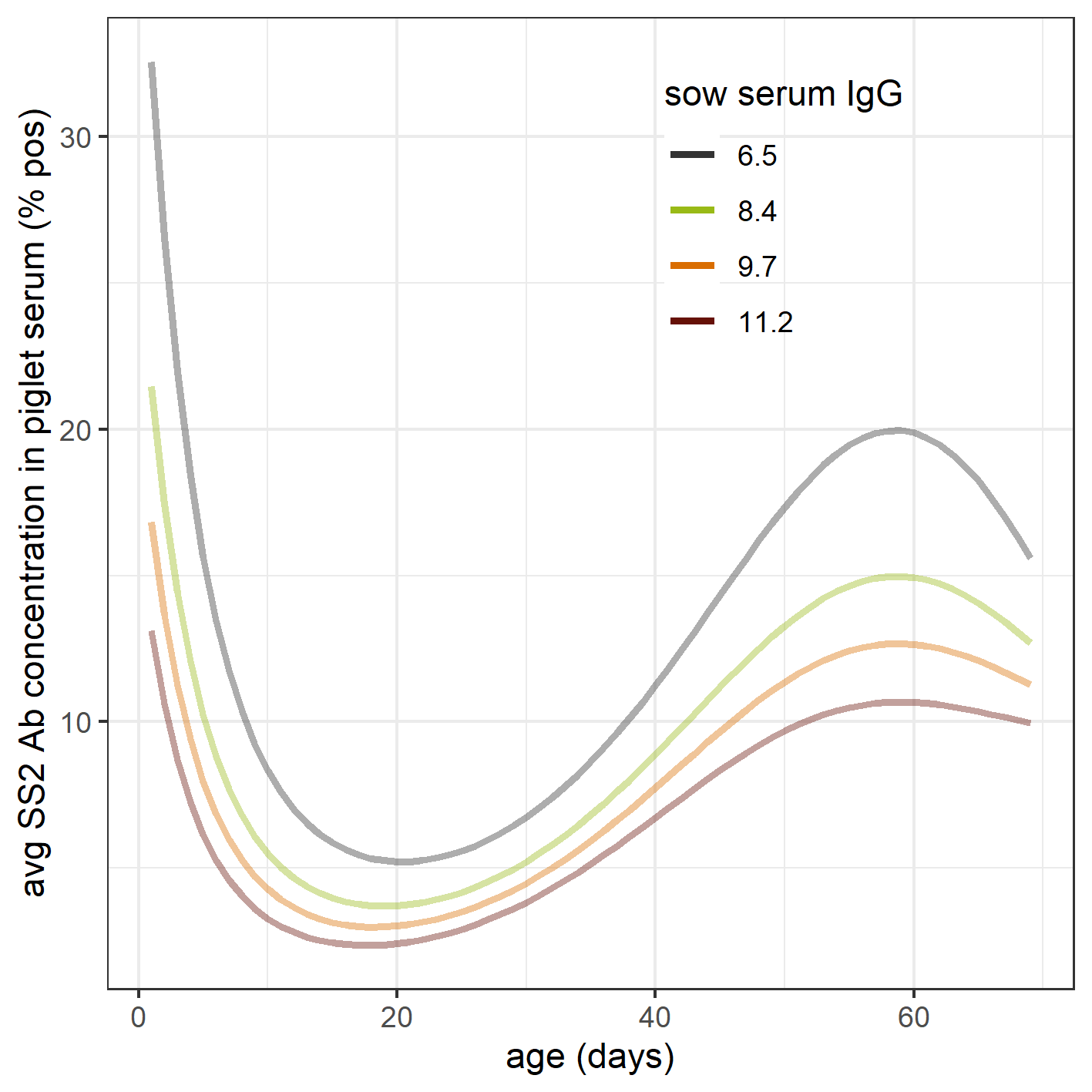 |
| 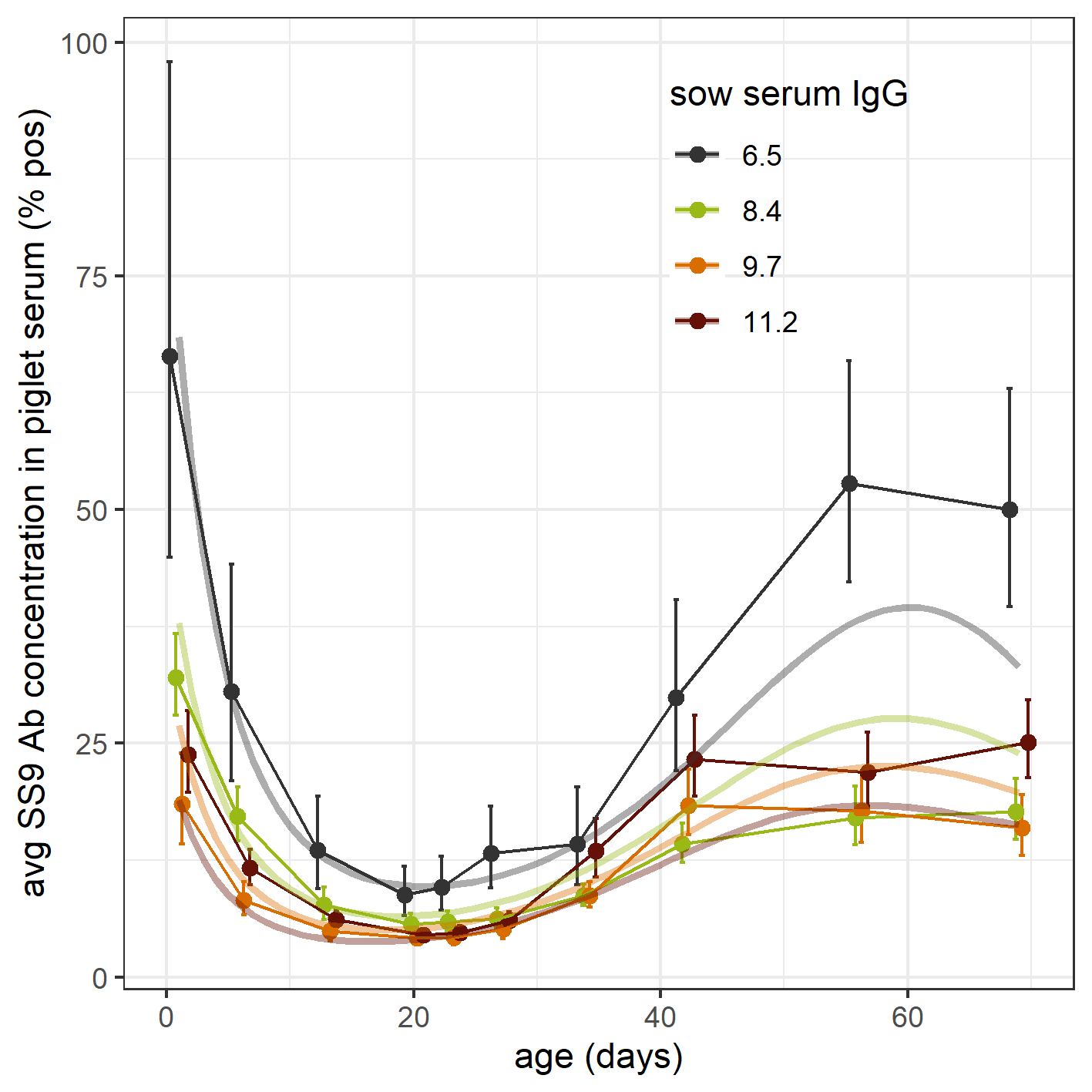 | 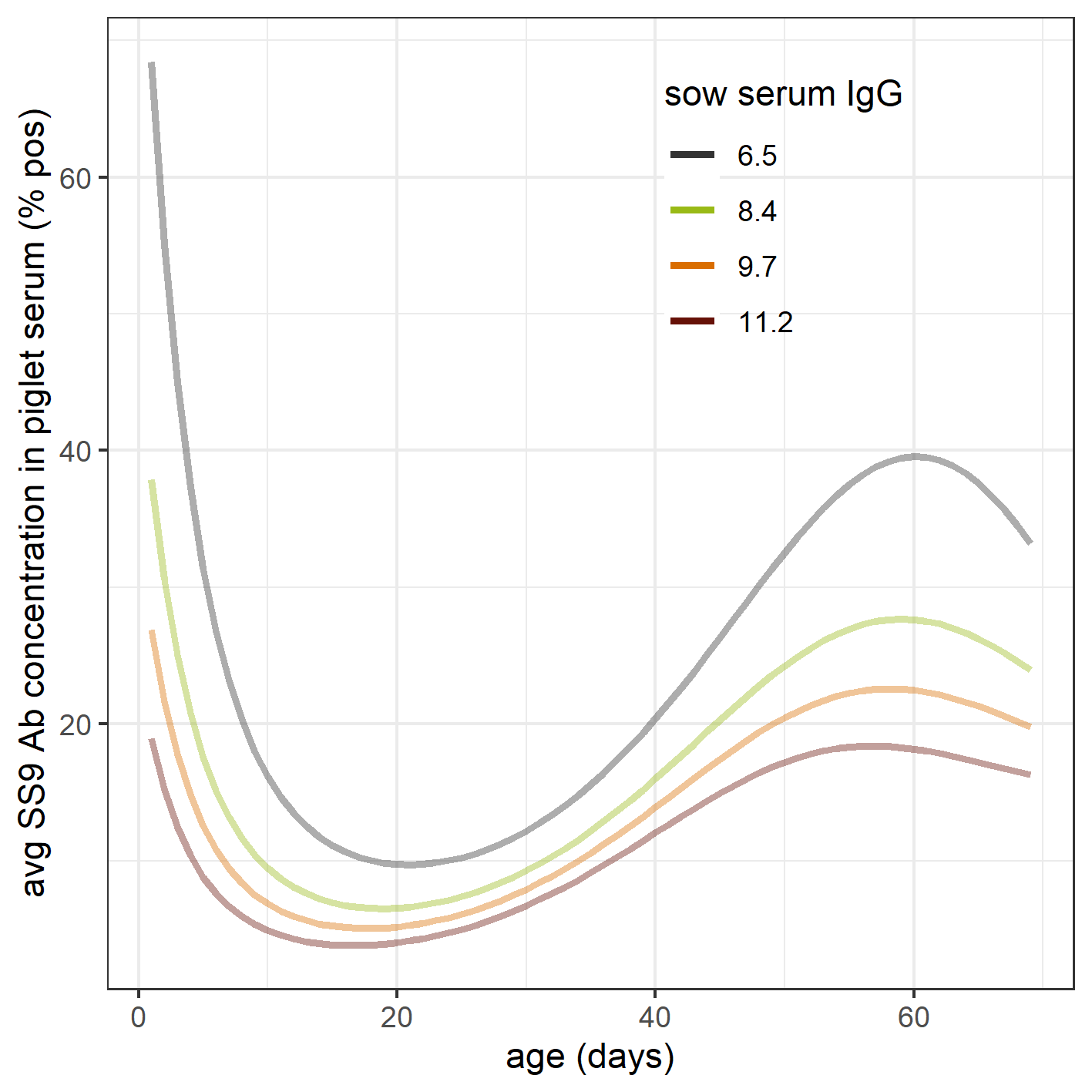 |

**G**: **Sow serum IgG** - The left graphic depicts four groups with different sow serum IgG levels and relates these levels to the % positive (pos)for *S. suis* specific antibodies for serotype 2 (Ss2, first row) and serotype 9 (Ss9, second row). For all ten blood sampling time-points the standard error of each group is shown. The right graphic depicts only the modelled version.
